# Determination of Binding Constants and Gas Phase Stabilities of Artificial Carbohydrate Receptor Complexes Using Electrospray Mass Spectrometry

**DOI:** 10.1021/acsomega.4c06976

**Published:** 2024-10-31

**Authors:** Alexander Weiß, Manuel Dutschke, Carla Vogt, Jan Zuber

**Affiliations:** † Institute of Analytical Chemistry, TU Bergakademie Freiberg, Lessingstraße 45, Freiberg 09599, Germany; ‡ MFPA Weimar—Materials Research and Testing Institute Weimar, Coudraystraße 9, Weimar 99423, Germany

## Abstract

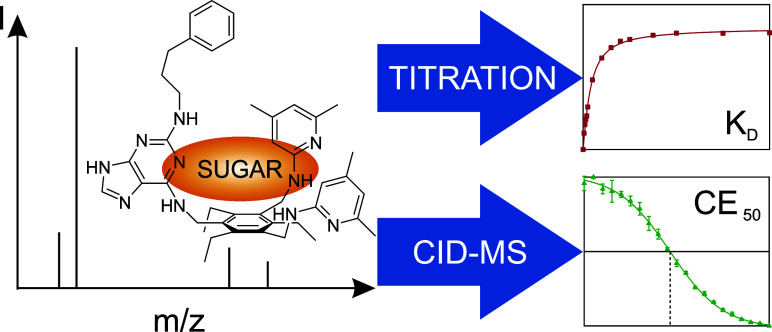

In recent years, binding studies to determine complex stabilities and selectivities of artificial carbohydrate receptors with glycosides have been mainly performed using ^1^H NMR, isothermal titration calorimetry (ITC), and other spectroscopic titration techniques. Native electrospray ionization (ESI) mass spectrometry is used only to verify the complex stoichiometries, although determination of dissociation constants is also possible. Herein, the binding of a 1,3,5-substituted 2,4,6-triethylbenzene-based receptor (CHR) to four alkyl-β-d-glucosides with varying alkyl side chain lengths (methyl (MGP), hexyl (HGP), octyl (OGP), and dodecyl (DGP)-β-d-glucosides), which was analyzed by ESI Fourier transform ion cyclotron resonance mass spectrometry (ESI-FT-ICR-MS) under optimized spray conditions in both ion modes, is reported. The complexes of the receptor with different sugars could be detected in 1:1 and 2:1 stoichiometries. Dissociation constants calculated for the 1:1 complexes showed a stability trend depending on the length of the alkyl side chain of the sugar: CHR:DGP > CHR:OGP > CHR:HGP > CHR:MGP. Gas phase stabilities determined by CID-MS confirm this relative trend in binding affinities. These findings substantiate the validity and applicability of ESI-MS as a method for investigating noncovalent complex stabilities and thus support research in the field of molecular recognition of carbohydrates by artificial receptors.

## 1 Introduction

Carbohydrate−protein interactions are found to play a key role in numerous biological recognition processes^1,2^ that take place on the surface of cells. They are important for a variety of physiological and pathological cell functions, such as antigen−antibody reactions, cell−cell or cell−matrix interactions, fertilization of egg cells, or immune responses.^3,4^ Despite their important roles, the details of these molecular interactions are not well-understood to this day and the subject of great research efforts.^5−7^ Based on X-ray crystallographic analyses, it is assumed that mainly hydrogen bonds, CH−π interactions, and van der Waals forces are involved in the recognition process of carbohydrate-binding proteins.^4^ To understand highly complex biological processes and associated interactions, selectively binding artificial carbohydrate receptors can also be used, which act as model systems and mimic the binding motifs in the carbohydrate−protein complexes.^8^ In addition to their model function, they could potentially also be applied as pharmaceuticals (e.g., anticancer or anti-infective or drug delivery agents)^1,9^ or biosensors (e.g., agents for disease detection),^10^ because of their biological activities.^11^

In recent years, a significant number of such artificial receptors have been synthesized.^12^ A subclass of these are the acyclic 1,3,5-substituted 2,4,6-triethylbenzene derivatives bearing different types of heterocyclic units and also purine^13^ units as recognition groups. They show interesting sugar-binding efficiencies and selectivities depending on the basic aromatic scaffold, the nature of the recognition groups, and the link between the aromatic structure and the recognition units. The acyclic scaffold offers an easy way to modify these recognition units during synthesis.^14,15^ Systematic structural modification of these purine-containing compounds enables the investigation of new structure−activity relationships and thus the development of new artificial sugar receptors with predictable binding properties, what has not yet been realized.^10^

For these investigations, binding studies are often carried out in organic media to determine the affinities in form of association (*K*_A_) or dissociation constants (*K*_D_) between the receptor−sugar complexes.^16^ In addition, the comprehensive characterization of the structure as well as thermodynamic and kinetic parameters also plays a role in the investigation of noncovalent interactions during sugar binding by artificial receptors.^17^ Biophysical methods including nuclear magnetic resonance (NMR) and “gold standard” isothermal titration calorimetry (ITC)^18^ are widely used to study the host−guest binding via titration experiments and to understand how structural variations affect binding constants and selectivities. The *K*_D_ ranges for the methods mentioned are in the range of 10−10^6^ μM for NMR^19^ and 10^−3^ to 10^3^ μM for ITC.^20^ However, both methods require the use of models to determine the complex stoichiometries present and are both time- and sample-consuming. Furthermore, they are less sensitive.^21^

In contrast, native ESI mass spectrometry is used only in investigations to determine the presence of weakly bound complexes in the gas phase or their stoichiometry. Of course, direct analysis of complex stoichiometries and multiple binding equilibria simultaneously is a great advantage for studying noncovalent host−guest binding.^22−24^ However, relative binding affinities can also be determined very quickly with very low sample consumption due to the high sensitivity of the method. Furthermore, native ESI mass spectrometry is capable of determining *K*_D_ values over a wide dynamic range (10^−3^ to 10^5^ μM).^17^ In addition, gas phase technologies such as collision-induced dissociation offer the possibility to probe the gas phase structures and stabilities of the noncovalent complexes. Collision energies CE_50_ for 50% dissociation of the complex can be used as a rapid achievable measure of complex stability.^25−28^

**Figure 1:**
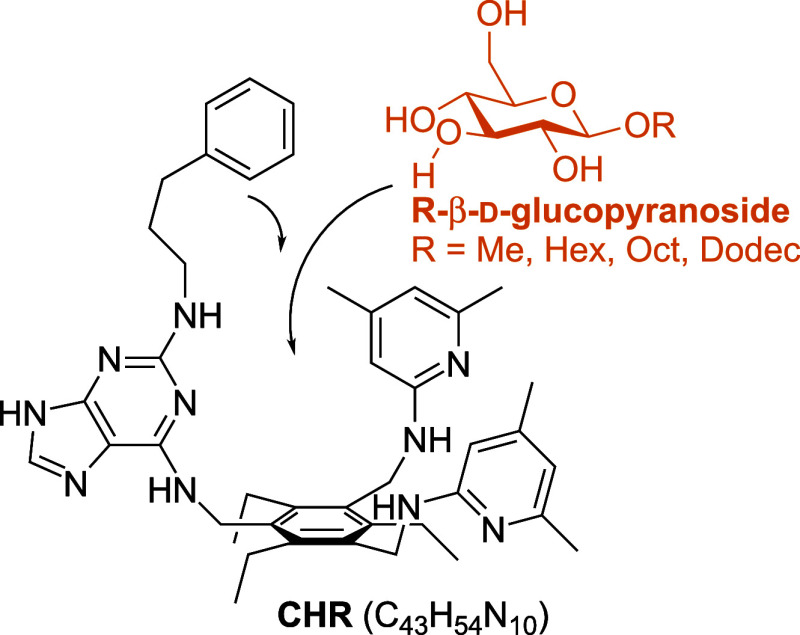
Schematic illustration of the binding of alkyl-β-d-glucopyranosides by carbohydrate receptor 1-[2-(phenylpropylamino)-9*H*-purin-6-yl]aminomethyl-3,5-bis-[(4,6-dimethylpyridin-2-yl)aminomethyl]-2,4,6-triethylbenzene (CHR). The glucosides are recognized via purine and dimethylpyridine recognition groups.

The aim of this study was to demonstrate that electrospray Fourier transform ion cyclotron resonance mass spectrometry (ESI-FT-ICR-MS) can be used as an analytical tool to perform supramolecular titrations and determine binding affinities for noncovalent complexes of artificial carbohydrate receptors and glycoside guest molecules. First, however, the mass spectrometric methods for nondestructive transfer of the supramolecular complexes into the gas phase had to be developed and carefully optimized for optimum source conditions and signal intensity. The importance of precise adjustment of the instrumental source parameters to obtain the weakly bound complex has already been described by Pedro et al.^29^ The artificial receptor 1-[2-(phenylpropylamino)-9*H*-purin-6-yl]aminomethyl-3,5-bis-[(4,6-dimethylpyridin-2-yl)aminomethyl]-2,4,6-triethylbenzene (CHR, Figure 1) used in our experiments has already been investigated in the past by the Mazik group^14,15^ for its affinity to octyl-β-d-glucoside (OGP) using ^1^H NMR spectroscopy. A pictorial representation of the binding of the receptor to glucosides is shown in Figure 1. Stability constants were determined for both the 1:1 and 2:1 complexes (CHR:glucoside).

In a manner equivalent to NMR titrations, we have now quantitatively determined the host−guest association by ESI titrations not only for OGP but also for three further alkyl-β-d-glucosides (methyl, hexyl, and dodecyl), in combination with further investigations on gas phase stabilities by collision-induced dissociation mass spectrometry (CID-MS). Since many noncovalent complexes in the literature have only been investigated with the negative ion mode,^30^ we wanted to provide the most comprehensive results possible by using both ion modes from the outset. This also allows both ion modes to be compared with each other. This is particularly important, as the receptor is both easily protonated and deprotonated because of its functional groups.

For the first time, complexes based on receptor molecules of tripodal form of this type have also been investigated by using ESI mass spectrometry to determine dissociation constants *K*_D_ and gas phase stabilities CE_50_. We hope that our methods will contribute to research into other artificial carbohydrate receptors and their binding affinities and selectivities in the future.

## 2 Experimental Section

### 2.1 Materials

Methyl-β-d-glucoside (MGP) was purchased from J&K Scientific (USA). Hexyl- and octyl-β-d-glucoside (HGP and OGP) were purchased from Sigma-Aldrich. Dodecyl−β-d-glucoside (DGP) was purchased from Glentham Life Sciences (UK). Carbohydrate receptor 1-[2-(phenylpropylamino)-9*H*-purin-6-yl]aminomethyl-3,5-bis-[(4,6-dimethylpyridin-2-yl)aminomethyl]-2,4,6-triethylbenzene (CHR) was synthesized by the Mazik group (Institute of Organic Chemistry, TU Bergakademie Freiberg, Germany) according to the instructions of Kaiser et al.^14,15^ and kindly provided to us. All substances were used without further purification. ^1^H and ^13^C NMR spectra of these compounds were recorded with a 500 MHz Bruker Avance III spectrometer and can be found in Supporting Information (see Section S1). Methanol (MeOH) was purchased from Chemsolute, acetone was purchased from VWR Chemicals, and dichloromethane (DCM) was purchased from Carl Roth. All utilized solvents were of HPLC grade quality. Deuterated solvents chloroform-*d* (99.8 at. % D) and deuterium oxide (99.8 at. % D) were bought from Armar Chemicals.

### 2.2 Sample Preparation

The stock solutions were freshly prepared on each measurement day. Glucoside samples and CHR were weighed into amber glass vials and then dissolved in the respective solvent to a concentration of 1 mmol/L. For the initial optimization experiments, methanol, DCM, and acetone were tested as solvents. In all of the further experiments, only methanol was used as a solvent. The solutions were sonicated for at least 10 min and stored refrigerated (5 °C) and out of the light when not in use. Mixtures of glucoside and CHR with an equimolar concentration of 5 μM were typically used for the development of analysis routines and collision-induced dissociation (CID) experiments. For the titration experiments, 1 mL sample solutions were prepared per concentration step. To the defined amount of methanol, 5 μL of the stock solution of the receptor (corresponding to 5 μM guest, [G]) and an increasing amount of the stock solution of the respective glucoside were added with each concentration step to avoid dilution effects. A total of 15 concentration steps were prepared, varying the sugar concentration (host, [H]) in the range from 0 to 200 μM (corresponding to host:guest ratios 0:1 to 40:1). Incubation time was 10 min at room temperature (20 °C).

### 2.3 Mass Spectrometry (FT-ICR-MS)

All MS experiments were performed on 15 T solariX FT-ICR-MS from Bruker Daltonics, equipped with an ESI source and operated in positive and negative ion modes, with a scan range from 153.50 to 2000.00 Da. Sample solutions were introduced using the integrated syringe pump at a typical flow rate of 10 μL/min, starting 15 min before the actual recording of the mass spectra, in order to equilibrate the system sufficiently. ESI settings in both ion modes were chosen from the results of a method development process for the ESI-FT-ICR-MS analyses, which is described in general in the “Results and Discussion” section. For the positive ion mode, the capillary voltage was set to −4400 V. An end plate offset of −500 V, a nebulizer/sheath gas pressure of 1.0 bar, a dry gas/counter flow gas flow of 2.0 L/min, and a dry gas temperature of 70 °C were used. For ESI(−)-FT-ICR-MS analyses, the capillary voltage was set to 3511 V. An end plate offset of −500 V, a nebulizer/sheath gas pressure of 1.0 bar, a dry gas/counter flow gas flow of 2.0 L/min, and a dry gas temperature of 237 °C were utilized. To avoid contamination between runs (memory effect), the ESI needle was washed with a solvent mixture consisting of 80% (v/v) MeOH and 20% (v/v) DCM for 5 min and with a flow rate of 0.25 mL/min. The ion accumulation time *t*_acc_ was adjusted for each of the sample solutions for optimal sensitivity, and the specific values can be found in Supporting Information (Section S2). Hexapole flight time *t*_F_ settings were made depending on the complex system studied (Table 1). Resulting data sets had a transient size of 8 M, while 32 scans (three determinations) were accumulated for each analysis. The resolving power was *R* = 800000 at *m*/*z* = 400 Da for all analyses.

CE_50_ values were derived through collision-induced dissociation studies using basically the same MS parameters that were used for all other analyses. Q1 mass was set to 170 Da. The complete amount of ions was accelerated to a determined kinetic energy (*E*_lab_: 0−10 eV) and guided into the collision cell filled with argon gas. Fragment ions as well as nonfragmented precursor ions were mass-analyzed in the ICR cell upon increasing the collision energy. Set values for *t*_acc_ and *t*_F_ can be found in Table 1.

**Table 1:** Ion Accumulation Time *t*_acc_ and Hexapole Flight Time *t*_F_ Settings Used for ESI-CID-FT-ICR-MS Analyses

host:guest	*t*_acc,+_ (s)	*t*_acc,−_ (s)	*t*_F_ (ms)
CHR:MGP	0.075	0.003	0.7
CHR:HGP	0.045	0.001	0.8
CHR:OGP	0.055	0.001	0.9
CHR:DGP	0.060	0.001	1.0

### 2.4 Data Processing

All recorded mass spectra were calibrated using in-house internal calibration lists within Bruker Daltonics software DataAnalysis 5.0 (SR1) and with a standard deviation of less than 0.2 ppm. The resulting peak lists were exported and imported into Matlab 2021b (Mathworks) before being processed further using in-house scripts for filtering and extracting peak intensities for calculated molecular formulas. Response surface plots were created with the help of STATGRAPHICS Centurion 18 software.

To determine CHR:glucoside *K*_D_’s by titration, the absolute intensities of bound receptor *I*_HG_ to total receptor *I*_G_ + *I*_HG_ (hereafter also referred to as Δ*I*_r_ or relative complex intensity) in the mass spectra were correlated to the equilibrium concentration of the bound receptor in the noncovalent complex [HG], with *k* as a proportionality constant. The constant *k* contains the nonuniform ESI response factors because of different ion sizes and solvation energies^31^ without their absolute values having to be known^32−34^
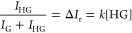
1Assuming that the concentration of free sugar [H] is equal to the total concentration of the sugar [H]_0_ (which is only valid for a high excess of [H]_0_ compared to [G]_0_), Dotsikas and Loukas^34^ proposed a Benesi−Hildebrand-like, double reciprocal linear equation, which correlates the relative complex intensity and the initial concentrations of the host and guest, respectively, glucoside and receptor in our case
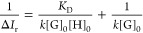
2Without any assumptions, and by plotting experimentally determined Δ*I*_r_ values against total host concentration [H]_0_, *K*_D_ could be obtained as a parameter of a nonlinear least-squares curve fitting. Simplification and multiple transformations (see Section S3 for details) of the equation given in the literature^34^ lead us to the following relation:
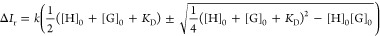
3*K*_D_ and *k* values were determined by using eq 3 and a self-written function in OriginPro 2019b, whereby the incorrect solution is discarded in each case. The singly protonated or deprotonated molecular ions ([M + H]^+^ for the positive ion mode or [M − H]^−^ for the negative ion mode, respectively) of the guest and complex were used for the evaluation because they are the most abundant ion types that occurred in all mass spectra.

To determine the CE_50_ values, mass spectra were recorded for different collision energies, and the relative intensity (RI) of a surviving precursor ion at each collision energy was calculated from the absolute intensities of the complex *I*_HG_, host/sugar *I*_H_, and guest/receptor *I*_G_ according to eq 4.^28^
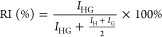
4Then, CE_50_ values were derived from plots of RI vs CE (= *E*_lab_) by using a fit with a sigmoidal Hill function (DoseResp function in OriginPro 2019b). Protonated and deprotonated forms as well as sodium adducts of the complexes were studied as precursor ions.

## 3 Results and Discussion

### 3.1 Development and Optimization of Analysis Routines

Despite the gentle transfer of the noncovalent complex from the liquid phase to the gas phase using ESI−MS in most cases, improperly selected ESI conditions can influence the signal intensity or, even more fatal, lead to the dissociation of the complex. For this reason, careful selection of the ESI conditions is essential when developing and optimizing new analysis routines.^29,35^ Our development and optimization process started with the ESI(+)-MS experiments. Initially, only the CHR:OGP complex system was analyzed because of its already known characterization with NMR spectroscopy.^14,15^ For the experiments, a solution of each component was prepared in the solvents methanol, dichloromethane, and acetone at a concentration of 5 μmol/L. The solvents were chosen for their different polarity and simultaneous applicability for MS experiments. Subsequently, optimal ionization parameters for a maximal absolute complex ion abundance were investigated with the help of a statistical design of experiments (DOE) plan in the Box−Behnken design.^36,37^ According to previously published results,^38^ it makes the most sense for robust and reproducible ESI-MS methods to optimize capillary voltage, dry gas flow, and dry gas temperature. The ratio of the intensities of bound to free guest or the relative complex intensity was also considered as response variables, but the same optimal ionization parameters were found as in the optimization for maximum absolute complex ion abundance.

Based on previous screening experiments, the experimental design was carried out in two stages. First, the effect of three factors was analyzed in 15 experiments, using the following [−1, 0, 1] tuples: capillary voltage |−|[3000, 3700, 4400] V, dry gas flow [1.5, 2.5, 3.5] L/min, and dry gas temperature [70, 135, 200] °C for methanol and capillary voltage |−|[4100, 4400, 4700] V, dry gas flow [2, 4, 6] L/min, and dry gas temperature [70, 135, 200] °C for acetone. Response surface and standardized Pareto plots are depicted in Figure S11 and S13, respectively. It was found that the capillary voltage has a significant influence and must be set as high as possible for maximal complex intensity in the mass spectra. However, higher voltages showed irregularities in the shield voltage. When using methanol, a dry gas flow of 3.5 L/min and a dry gas temperature of 200 °C are ideal to achieve the highest abundance for CHR:OGP. For the use of acetone, the optimum values are 2.7 L/min and 70 °C. No results could be obtained for dichloromethane as CHR:OGP was not detectable. The low boiling point of the solvent^39^ causes it to evaporate too quickly, leading to short-term changes in the concentrations of the complex partners that can alter the equilibrium and destroy the complex.^40^

In a second step, a 3^2^ factorial design plan with 9 experiments was carried out, which analyzes the effect of two factors (dry gas flow and dry gas temperature, capillary voltage was held at maximum values) in the range of 2−6 L/min and 70−200 °C, respectively. The parameters should be set to the lowest possible values for both solvents (−1/−1). This also corresponds to the technical limitations of the device. If the entire response surface (Figures S12 and S14) is considered, it is also noticeable that the two parameters also produce almost optimum intensities at factor values of +1/+1. A high dry gas flow has the negative consequence that many cations generated in the ESI source are deflected from their path into the analyzer and are discharged at the cathode. The high dry gas temperature can counteract this, as it enables better drying of droplets and therefore a higher number of free analyte ions. The optimized signal intensities for the [M + H]^+^ of the CHR:OGP complex in methanol are 3.6 × 10^8^ counts and in acetone 4.98 × 10^7^. Therefore, methanol was chosen as solvent for further experiments.

**Figure 2:**
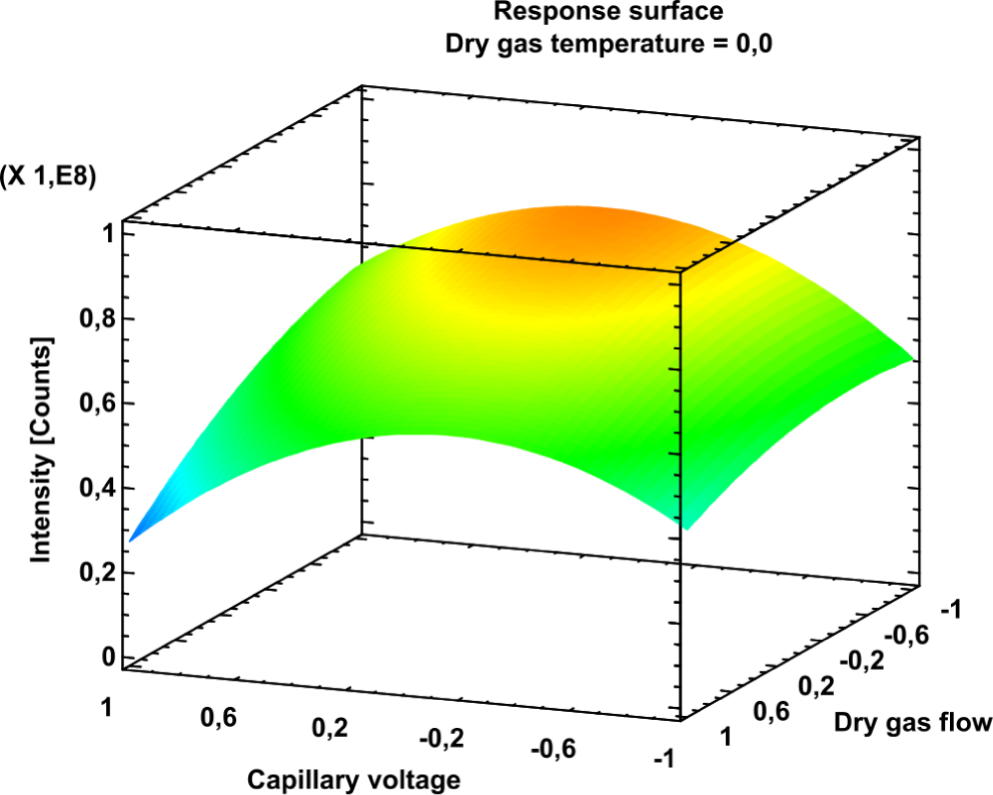
Resulting response surface of the DOE plan in the Box−Behnken design, which was used to optimize the ESI source conditions. Complex ion abundances (for [M − H]^−^ molecular ion) are presented color-coded (blue: low intensity; green: medium intensity; red: high intensity).

The optimization process for the negative ion mode started with the prerequisites and results from the positive ion mode. The resulting response surface from the DOE optimization of capillary voltage (3400−3600 V), dry gas flow (2.0−8.0 L/min), and dry gas temperature (70−350 °C) is presented in Figure 2. It was revealed that a capillary voltage of 3511 V, a dry gas flow of 2.0 L/min, and a dry gas temperature of 237 °C are ideal to achieve the highest abundance for the [CHR:OGP-H]^−^. Here too, the setting for the dry gas flow corresponds to the lowest possible setting. It was necessary to adapt the spray conditions for both ion modes, as it was not possible to achieve maximum abundances for the respective complex ions when applying the optimized values to the other ion mode. Despite different spray conditions, there is still comparability between both ion modes, as the literature also shows.^30,45^

### 3.2 Mass Spectral Results

**Figure 3:**
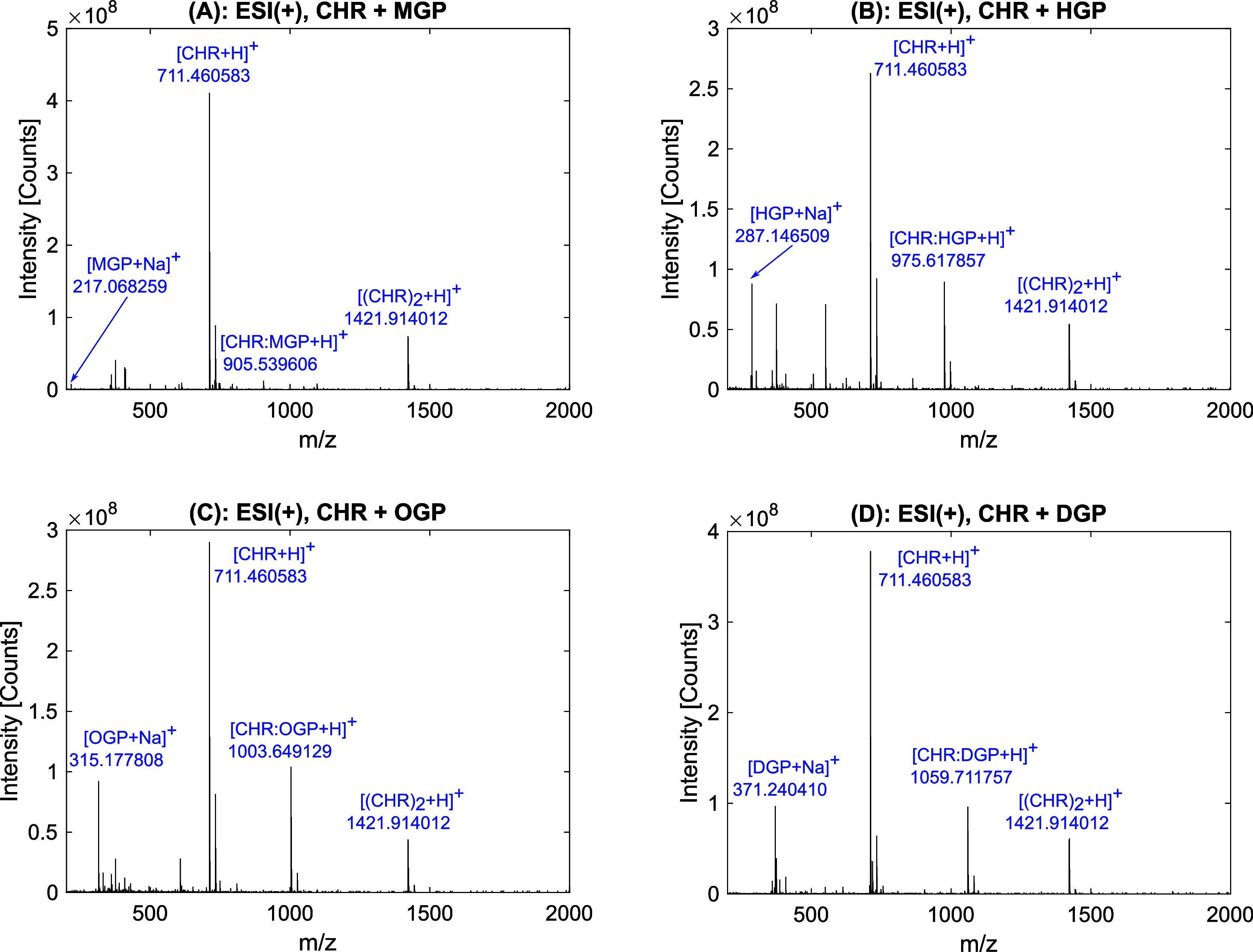
Positive-ion ESI mass spectra for the mixture of (A) CHR and MGP, (B) CHR and HGP, (C) CHR and OGP, and (D) CHR and DGP. The concentrations of both CHR and the respective glucoside were 5 μmol/L. Selected molecular ions are labeled and indicated with their corresponding, theoretically calculated exact masses. For further exact masses of other molecular ions, see Tables S3 and S4 in Supporting Information. These masses were used to calibrate the mass spectra.

The 1:1 and 2:1 stochiometric complexes found in the liquid phase^14^ can also be found in the gas phase ESI-MS experiments. Obtained and processed mass spectra of the equimolar CHR:glucoside complex systems ([H]_0_ = [G]_0_ = 5 μmol/L) in positive ion mode are shown in Figures 3A−D. The most abundant molecular ions from the ESI(+) and ESI(−) mass spectra with their respective masses and assigned ion formulas are given in Tables S3 and S4.

In the positive mode, the main peak is always the peak of the H^+^ adduct of CHR (*m*/*z* 711.460583). The peak at *m*/*z* 733.442535, which, however, is much less intense, represents the Na^+^ adduct of CHR. The receptor can also be observed in the dimerized forms [(CHR)_2_ + H]^+^ and [(CHR)_2_ + Na]^+^ at *m*/*z* 1421.914012 and 1443.895775, respectively. In general, the most abundant ions in the spectra are singly protonated, but there are also less abundant Na^+^ adducts. Only for CHR is there also a peak for a doubly charged ion (*m*/*z* 356.233922). The high abundance of receptor and complex ions is due to the very good protonation capacity of the nitrogen-containing functionalities of CHR. In contrast, the glucoside used in each case cannot be protonated at all. The peaks at *m*/*z* 217.068259, 287.146509, 315.177808, and 371.240410 are the ions of Na^+^ adducts of MGP, HGP, OGP, and DGP, respectively. These glucosides are also present as dimers (*m*/*z* for MGP: 411.147297, HGP: 551.303798, OGP: 607.366441, and DGP: 719.491598). In addition to the protonated and Na^+^ adduct forms (*m*/*z* for MGP: 905.539606 and 927.521551, HGP: 975.617857 and 997.599801, OGP: 1003.649129 and 1025.631060, and DGP: 1059.711757 and 1081.693701) for the 1:1 complexes, the peaks for the 2:1 complexes are also visible in the spectra (MGP: *m*/*z* 1615.992898 and 1637.974842, HGP: *m*/*z* 1686.071149 and 1708.053093, OGP: *m*/*z* 1714.102449 and 1736.084393, and DGP: *m*/*z* 1770.165049 and 1792.146993). However, these signals are significantly less abundant.

In the negative ion mode, less abundant molecular ions of the form [M + Na − H_2_]^−^ are present in addition to the dominant singly deprotonated molecular ions. The base peak represents a single deprotonated carbohydrate receptor (*m*/*z* 709.446015). This can be attributed to the fact that the amidine-like functional groups in the two pyridine units and the purine unit are easily deprotonated and the deprotonated forms are mesomerically stabilized. The dimer of the receptor can also be observed with low abundance (*m*/*z* = 1419.899307). The peaks for the singly deprotonated glucosides (*m*/*z* for MGP: 193.071762, HGP: 263.150012, OGP: 291.181312, and DGP: 347.243912) again show a much lower intensity compared with the receptor. Here, the functional groups are difficult to deprotonate. A major part of the ionized glucosides is more likely to be found as dimers (*m*/*z* for MGP: 387.150800, HGP: 527.307301, OGP: 583.369901, and DGP: 695.495101). The ions at *m*/*z* 903.525054, 973.603304, 1001.634604, and 1057.697204 are [CHR:MGP-H]^−^, [CHR:HGP-H]^−^, [CHR:OGP-H]^−^, and [CHR:DGP-H]^−^, respectively. The ions at *m*/*z* 1613.978345, 1684.056596, 1712.087896, and 1768.150496 represent the 2:1 complexes. As in the positive ion mode, their peaks are less intense compared to the 1:1 complex peaks.

### 3.3 CHR:Glucoside Titration Experiments and *K*_D_ Determination

In recent years, various approaches have been proposed to determine binding constants and affinities of noncovalent complexes by titration experiments using native ESI-MS.^17,26,41−47^ Some approaches also rely on the use of an internal standard. Our initial experiments with selected standard compounds such as long-chain perfluoroalkanoic acids, tetracyclines, and nucleotides, which have structural similarities to the complex systems investigated, had shown that quantification to determine the proportion of free host [H] using external calibration is not possible. Thus, the complexation behavior and ionization of CHR and the OGP used for the test experiments were negatively influenced by suppression effects. The addition of the standard, as well as the variation of the CHR concentration (>5 μmol/L) at a constant concentration of the sugar, meant that reproducible peaks could no longer be observed for either the sugar or the complex. Simplified approaches for the direct use of peak intensities instead of concentrations, assuming equal ionization efficiencies and uniform ESI response factors (RFs) for one of the complex partners and the complex,^31,48^ were also rejected because the difference in masses, sizes, and surface areas between the receptor and complex or sugar and complex is too large.^23^ For these reasons, a standard-free titration procedure, which also does not require the experimental determination^24^ of the absolute or relative RF, was selected, where the data are fitted according to the equation derived by Dotsikas et al.^33,34^ Interestingly, the expression for [HG] (eq 3) that we have obtained from simplification and multiple transformations (see Section S3) corresponds to the equation proposed by Thordarson.^49−51^ This observation results in a simplification for the nonlinear regression and shows the comparability of NMR and ESI-MS titration approaches.

The concentration of glucoside was chosen as the titration variable because of the high ionizability of the carbohydrate receptor compared to that of weakly ionizable sugars. The receptor concentration [G] was kept constant at 5 μmol/L for the titration experiments, while the glucoside concentration [H] was varied (0−200 μmol/L). For CHR:OGP, the observed intensities of selected peaks for the positive and negative ion modes are shown in Figures S15 and S16, respectively. As the concentration of the glucoside was increased, the intensity of the complex *I*_HG_ (singly protonated molecular ion) also increased, while the intensity of the receptor *I*_G_ decreased. This applies to the [M + H]^+^, [M + Na]^+^, and the singly protonated dimer. However, the observed intensity changes in the cases of the complex and receptor did not occur to the same extent because, as described above, the receptor and complex differ in size and the associated ionization efficiency. In line with our theoretical expectations, the abundance of the sodium adduct of the sugar and its dimer also increases with an increasing OGP concentration. The sodium adduct of the complex also becomes increasingly less abundant with an increasing sugar concentration, as sodium ions tend to form adducts with the dimer of the sugar. For the 2:1 complex, peaks can only be observed up to a concentration of 10 μmol/L, sometimes only in certain individual analyses. On the one hand, an excess of receptor is present only up to this concentration, and on the other hand, the 2:1 complex appears to be considerably less stable even at this level than the 1:1 complex. This also corresponds to the result of Kaiser et al., who found a 35-fold smaller binding constant for the 2:1 complex using NMR titration.^14^ In the negative mode, a permanent increase in intensity can only be observed for the OGP dimer with increasing [OGP]. For high concentrations above 10 μmol/L (CHR:OGP ratio greater than 2:1), saturation appears to have been reached. This means that the intensity does not change with increasing concentration. This ion signal saturation can be attributed to insufficient ionization during the electrospray process as a result of competition for available charge carriers (formation of charged droplets).^34,52^ For the singly deprotonated OGP ion and the complex, an approximately equal increase can also be observed initially, but the intensities drop again from the concentration mentioned. The decreasing abundance for the receptor and its dimer corresponds to theoretical expectations. The 2:1 complex could not be observed. As the 2:1 complex occurred as a peak only in certain single analyses, as described above, no further evaluation could be performed. These observations can also be made to a similar extent for the other sugars used, as shown in Figures S17−S22.

**Figure 4:**
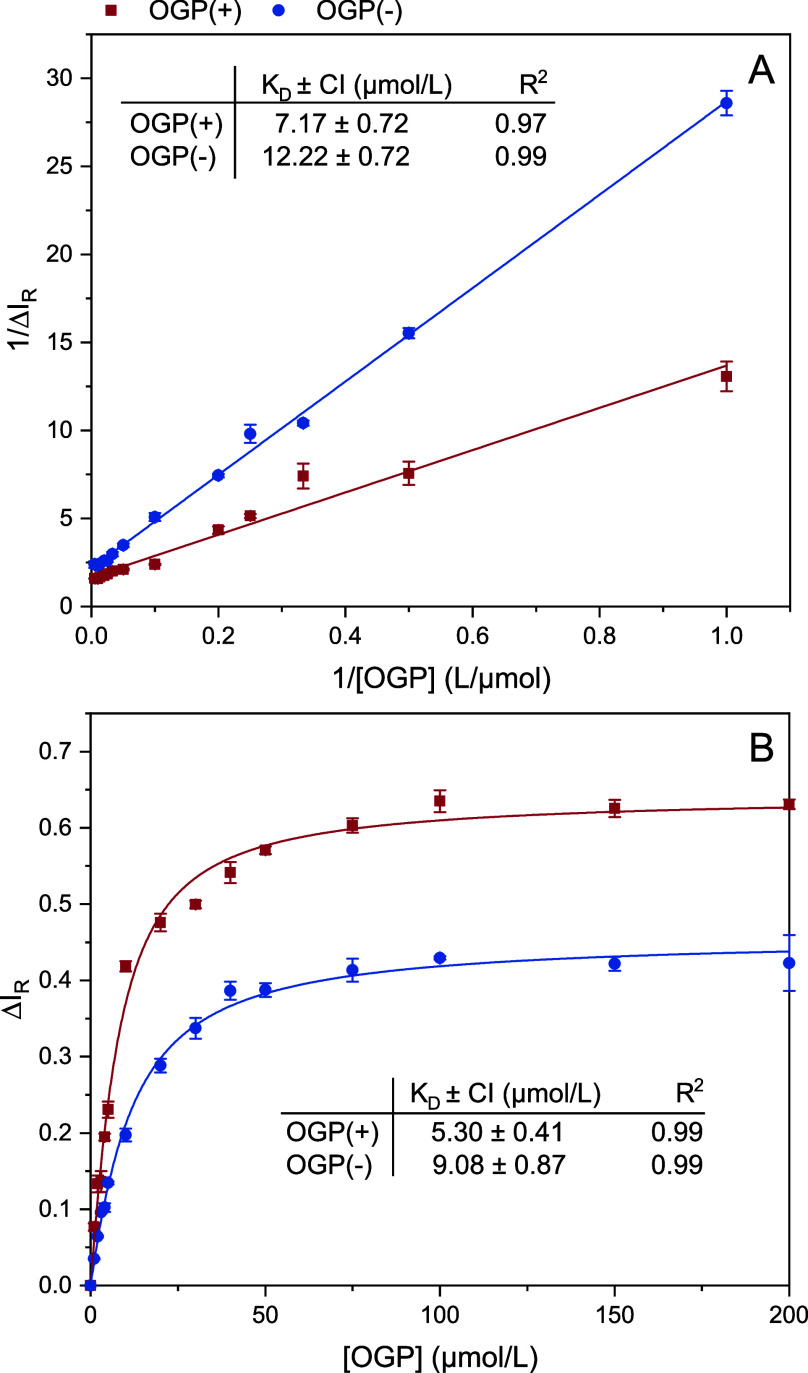
(A) Double reciprocal representation of the dependence of relative intensity Δ*I*_R_ on sugar concentration [OGP]. The data were fitted linearly, according to eq 2. (B) Titration curves for the CHR:OGP complex in positive and negative ion mode. The data were fitted according to eq 3. Error bars represent standard deviation (*n* = 3). The dissociation constants determined as well as their 95% confidence interval (CI) for triplicate analyses and the coefficient of determination *R*^2^ of the fit function are shown in the inset tables.

To determine the dissociation constants, the relative intensity of complex Δ*I*_R_ as a function of the glucoside concentration was plotted. Both double reciprocal and direct plotting of Δ*I*_R_ against [H] were used. Because of their behavior described above in relation to the altered glucoside concentration and their high abundance in contrast to other ions, the singly protonated or deprotonated molecular ions ([M + H]^+^ for the positive ion mode or [M − H]^−^ for the negative ion mode, respectively) of the receptor and complex were used to calculate Δ*I*_R_. Regressions were then carried out for the plotted values according to eq 2 (linear approach) and 3 (nonlinear approach). As an example, the linear and nonlinear plots for the CHR:OGP complex are shown in Figure 4A,B, together with the dissociation constants determined from the fits. The analog plots for the other sugars used can be found in Supporting Information (Figures S23−S25). All dissociation constants determined from the linear and nonlinear methods are summarized in Table 2. With one exception, the coefficients of determination *R*^2^ for the fits are all greater than or equal to 0.95, which indicates a good fit of the model to the experimental data. However, the results obtained from the linear approach (according to eq 2) and the nonlinear approach (eq 3) differ. This is because the assumption [H] = [H]_0_ must be made for the linear approach, which is actually only valid for high sugar surpluses. In our case, however, values in the range of CHR:glucoside ratios of 1:0−1:1 were also included in the calculations. The linear approach can be deliberately dispensed with further experiments, especially as the nonlinear adjustment is very easy to perform with the available evaluation programs. Furthermore, it is in line with our expectations that the dissociation constants for negative ion mode *K*_D−_ are higher compared to the values for positive mode *K*_D+_. The higher temperatures applied in the negative mode result in a lower stability of the noncovalent complex. However, a direct conversion of the values is not possible.

A certain trend in stability can be observed among the different complexes studied. In general, it can be said that the stability increases from the CHR:MGP to the CHR:DGP complex as the alkyl chain length on the glucoside increases. This alkyl chain normally represents the connection of the carbohydrate to the glycocalix^4^ on the outer surface of the cell membrane and therefore only plays, in a biological context, a subordinate role in the binding between the receptor and carbohydrate. We hypothesize that the increase in stability can be explained by additional van der Waals contacts and CH−π interactions between the sugars and the carbohydrate receptor. The fact that dispersion forces increase in the absence of surrounding solvents^26,28^ may also explain the general stability even at temperatures far above room or physiological temperature. For the CHR:OGP complex, the values were also compared with the *K*_D_ values determined by NMR spectroscopy in CDCl_3_ at 25 °C by Kaiser et al.^14^ These are in the range of 11.99−12.99 μM. Although our determined values do not agree with this, they are in the same order of magnitude range. It should also be noted that our measurements and the NMR measurements from the literature were determined at different temperatures while using also different solvents. This was necessary because there are solubility problems with chloroform when using other glycosides besides OGP. Avoiding this problem by adding other solvents (e.g., water) was not an option for us. We opted for a single-solvent system in which the receptor and all of the sugars can be analyzed under the same solvent conditions. Methanol as a solvent, in which all sugars and the receptor dissolve, was therefore the better choice.

The solutions were incubated at 20 °C before the measurements; complexation therefore also took place at this temperature. The temperatures given in Table 2 correspond to those of the dry gas and are thus the maximum temperatures at which the complexes can be briefly exposed during transfer through the ESI source. It cannot be completely ruled out that the different dry gas temperatures for the two ion modes have an influence on the thermodynamic equilibrium (as shown by the lower stabilities of the complexes at higher temperatures in the negative mode). The higher stability values, even at higher temperatures, are an indication that the interactions between the receptor and sugar increase in the gas phase. Influences of nonspecific binding (e.g., dimer formation of glucosides and also CHR) can also contribute to this increase. Furthermore, when considering the deviations between the values, it must be noted that the determined values for the positive and negative ion modes also only reflect the stabilities of the ion types under consideration. It is therefore logical to also specify a *K*_D_ value for both ion modes, even if there is only one thermodynamic dissociation constant in the solution.

**Table 2:** Summary of Computed Dissociation Constants, *K*_D_, for the CHR:Glucoside Complexes (CI-95% Confidence Interval for *n* = 3)^a^

	(μmol/L)^b^	*R*^2^	(μmol/L)^b^	*R*^2^	(μmol/L)^c^	*R*^2^	(μmol/L)^c^	*R*^2^
	linear		nonlinear		linear		nonlinear	
CHR:MGP	11.83 ± 1.23	0.90	16.16 ± 0.87	0.96	15.17 ± 3.67	0.96	29.25 ± 2.57	0.99
CHR:HGP	1.65 ± 0.05	0.99	5.38 ± 0.75	0.99	13.52 ± 3.62	0.98	9.83 ± 0.74	0.99
CHR:OGP	7.17 ± 0.72	0.97	5.30 ± 0.41	0.99	12.22 ± 0.72	0.99	9.08 ± 0.87	0.99
CHR:DGP	7.25 ± 0.36	0.95	2.43 ± 0.29	0.98	5.86 ± 0.07	0.99	4.44 ± 0.54	0.98

a Values from the linear and nonlinear approaches are given.

b Values determined at 70 °C.

c Values determined at 237 °C.

### 3.4 Collision-Induced Dissociation Studies

**Figure 5:**
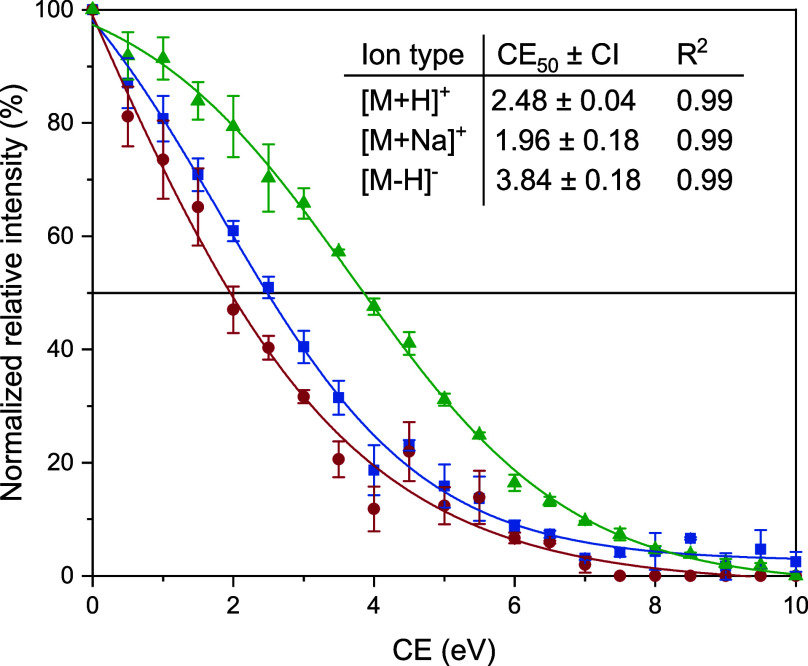
Normalized relative intensities of complex molecular ions [M + H]^+^ (blue), [M + Na]^+^ (red), and [M − H]^−^ (green) for CHR:OGP as a function of the collision energy (CE) obtained in the CID-MS experiments. Error bars represent the standard deviation for triplicate analyses. The CE_50_ value and the 95% confidence interval (CI) as well as the regression coefficient for the fit function are given for each breakdown curve.

Collision-induced dissociation studies were performed on the singly charged CHR:glucoside complexes [M + H]^+^, [M + Na]^+^, and [M − H]^−^. The fragment ions were the singly protonated ions or sodium adducts of the respective sugar and CHR in the positive ion mode and both singly deprotonated glucoside and receptor in the negative ion mode. The observed ion abundances of these ions depending on the collision energy used can be found in Supporting Information (Figures S26−S33). The intensity of the [M + H]^+^ fragment ions of glycosides was 0 over the entire energy range. Nevertheless, in order to calculate the CE_50_ values for [M + H]^+^ of the complex, we used the intensity of the sodium adduct of the respective glycoside in the calculations. These abundance values were used in order to calculate the normalized relative complex intensity of a surviving parent ion at each collision energy according to eq 4. Exemplary breakdown curves for the complex ions of the CHR:OGP complex resulting from these calculations are illustrated in Figure 5. Additional curves for the other complexes can be found in Figures S34−S36. To estimate the gas phase stability of ions, we used the collision energy at which half of the complex is dissociated (CE_50_). All of the determined stability values are summarized in Table 3. For all three complex ions investigated, the general trend can be observed that the stability of the complexes increases with increasing chain length of the alkyl chain on the glucoside. This finding also corresponds to the trend observed for the dissociation constants. However, the increase in the gas phase stabilities or the decrease in the dissociation constants with increasing alkyl chain length behaves differently for ions, and no mathematical correlation can be found. In particular, it is noticeable in the CE_50_ values that the values for [M − H]^−^ are significantly higher compared to the positively charged molecular ions of the complex. However, the stability for [M − H]^−^ derived from the dissociation constants is lower compared to that for positive ions. This inverse trend for the CE_50_ values cannot be explained by the temperature during the electrospray process. A possible explanation could be that the stability of the positively charged fragments is generally higher compared to that of the [M − H]^−^ fragments used for the negative ion mode for the evaluation. The influence of nonspecific aggregation cannot be ruled out here either.

Irrespective of this, the results of the dissociation experiments confirm the established trend for the stabilities of the CHR:glucoside complexes investigated and thus represent a supplement to the ESI titrations carried out. Since only one sample has to be prepared for the experiments and the analyses only consist of a continuous change of the collision energy, the CID studies offer a great time advantage and allow a quick estimation of the relative stabilities of structurally similar noncovalent complexes.

**Table 3:** Summary of Dermined Gas Phase Stabilities, CE_50_, for the Complex Ions of the Respective CHR:Glucoside Complexes (CI-95% Confidence Interval for *n* = 3)

	(eV)	*R*^2^	(eV)	*R*^2^	(eV)	*R*^2^
Ion type	[M + H]^+^		[M + Na]^+^		[M − H]^−^	
CHR:MGP	1.56 ± 0.19	0.99	1.05 ± 0.24	0.99	2.56 ± 0.02	0.99
CHR:HGP	2.19 ± 0.04	0.97	1.70 ± 0.09	0.97	2.85 ± 0.09	0.99
CHR:OGP	2.48 ± 0.04	0.99	1.96 ± 0.18	0.99	3.84 ± 0.18	0.99
CHR:DGP	3.11 ± 0.49	0.99	3.26 ± 0.40	0.92	4.63 ± 0.14	0.99

## 4 Conclusion

The development of artificial carbohydrate receptors with predictable binding properties and selectivities depends on the identification of structure−activity relationships and continues to be the subject of extensive research. The complexation behavior of potential receptors toward carbohydrates and the determination of dissociation constants have so far been investigated mainly by means of ^1^H NMR and other spectroscopic titration experiments. In the present study, the binding of a 1,3,5-substituted 2,4,6-triethylbenzene-based receptor (CHR) against various β-d-glucosides was used as an example to show that native ESI-MS is also a convenient way to successfully confirm and quantify the interaction between carbohydrate receptors and carbohydrates. For this purpose, two different ESI-FT-ICR-MS methods for positive and negative ion modes were developed and optimized in order to transfer the noncovalent complexes nondestructively from a methanolic solution into the gas phase at spray temperatures of 70 and 237 °C, respectively.

ESI-MS spectra of all receptor−sugar complexes revealed the presence of not only 1:1 receptor−glucoside complexes but also the 2:1 stoichiometry, which could also be detected in the liquid phase.^14^ Based on the dissociation constants determined by our standard-free ESI titration procedure, the general trend was observed that the binding affinity to the receptor increases with increasing chain length of the alkyl chain on the β-d-glucosides, which we attribute to additional CH−π interactions between the alkyl chain and the recognition groups of the artificial receptor. The complex stabilities of the investigated complexes behave accordingly as follows: CHR:DGP > CHR:OGP > CHR:HGP > CHR:MGP. Additional collision-induced dissociation studies carried out on selected complex ions to determine gas phase stabilities have confirmed this relative trend. However, a direct comparison of the absolute *K*_D_ values and CE_50_ values is not possible, as the *K*_D_ values are thermodynamic absolute values, which provide information about the thermodynamic equilibrium in solution, and the CE_50_ values are merely a semiquantitative measure of the stability of the complex ions in the gas phase.

Furthermore, no binding affinities could be determined for the 2:1 complex, as corresponding peaks for the complex in the mass spectra occurred only in a limited concentration range and were not always reproducible. Our approach of using both ion modes to study complex stabilities also shows that the stability values obtained reflect the ion stabilities. This is why different absolute values result for both ion modes—they simply depend on the respective ion type.

Further work will now apply the methods developed to other receptors and sugars, whereby ionization at other temperatures, such as room temperature, still needs to be optimized for better comparability. Furthermore, a correlation spectroscopic approach for ^1^H NMR and MS titration as well as theoretical data sets can help to cross-verify the determined dissociation constants. Precisely, because solvent effects do not play a role in mass spectrometric analysis, it represents a counterpoint to analyses in the solution phase and, therefore, provides additional information. Matching of solvents for NMR and MS could also give further information about the solvent-induced influence of nonspecific binding.

## Supporting Information Available

Liquid-state ^1^H and ^13^C NMR spectra of 1-[2-(phenylpropylamino)-9*H*-purin-6-yl]aminomethyl-3,5-bis-[(4,6-dimethylpyridin-2-yl)aminomethyl]-2,4,6-triethylbenzene (CHR) and *R*-β-d-glucosides; ion accumulation time settings used for ESI-MS titration experiments; derivation of expressions for calculating complex concentrations from mass spectral intensities; response surface and Pareto plots from the development and optimization of analysis routines; lists of most abundant molecular ions from mass spectra with calculated ion masses and assigned ion formulas; further results from ESI-MS titration experiments and determination of dissociation constants; additional results from CID-MS experiments and CE_50_ evaluation (PDF)
