# Supplementary material for: Determination of Binding Constants and Gas Phase Stabilities of Artificial Carbohydrate Receptor Complexes Using Electrospray Mass Spectrometry
Source: ACS Omega. 2024 Oct 31;9(45):45309–18. doi: 10.1021/acsomega.4c06976 (PMC11561607; doi:10.1021/acsomega.4c06976)
Supplement: Supplementary file 1 — ao4c06976_si_001.pdf [file ao4c06976_si_001.pdf]

SUPPORTING INFORMATION

Determination of Binding Constants and Gas Phase Stabilities of Artificial Carbohydrate Receptor Complexes Using Electrospray Mass Spectrometry

Alexander Weiß[a,*], Manuel Dutschke[b], Carla Vogt[a], and Jan Zuber[a,*]

[a]*Institute of Analytical Chemistry, TU Bergakademie Freiberg, Lessingstraße 45, 09599 Freiberg, Germany*
[b]*MFPA Weimar – Materials Research and Testing Institute Weimar, Coudraystraße 9, 99423 Weimar, Germany*
* *Corresponding Authors, E-mail: al.weiss@chemie.tu-freiberg.de; jan.zuber@chemie.tu-freiberg.de*

Contents

S1. NMR Spectra of Used Carbohydrate Receptor (CHR) and R-β-D-glucosides S-2

S2. Ion Accumulation Time t_{acc} Settings Used for ESI-MS Titration Experiments S-12

S3. Derivation of the Expressions for Calculating the Complex Concentration S-14

S4. Development and Optimization of Analysis Routines for ESI(+/-)-MS S-18

S5. Mass spectrometrical results S-20

S6. Further Results from ESI-MS Titration Experiments and Determination of Dissociation Constants S-23

S7. Further Results from CID-MS Experiments and CE_{50} Evaluation S-33

S1. NMR Spectra of Used Carbohydrate Receptor (CHR) and R-β-D-glucosides

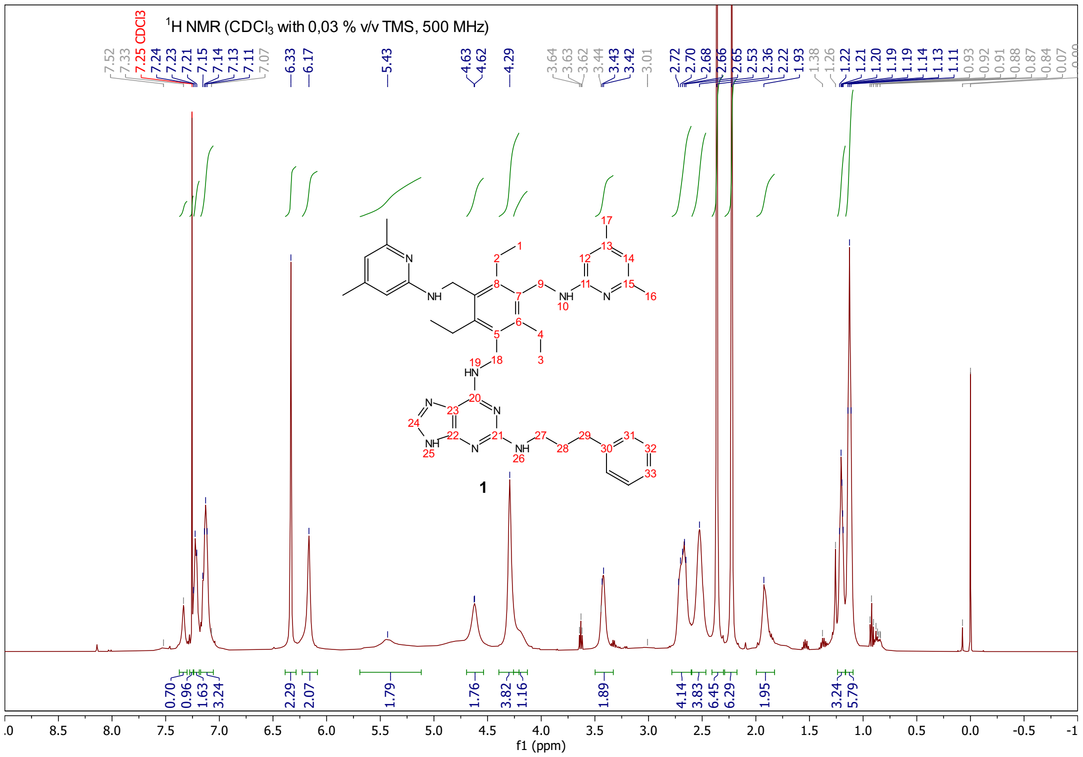

Figure S1: ^{1}H-NMR spectrum of 1-[2-(Phenylpropylamino)-9H-purin-6-yl]aminomethyl-3,5-bis-[(4,6-dimethylpyridin-2-yl)aminomethyl]-2,4,6-triethylbenzene (CHR).

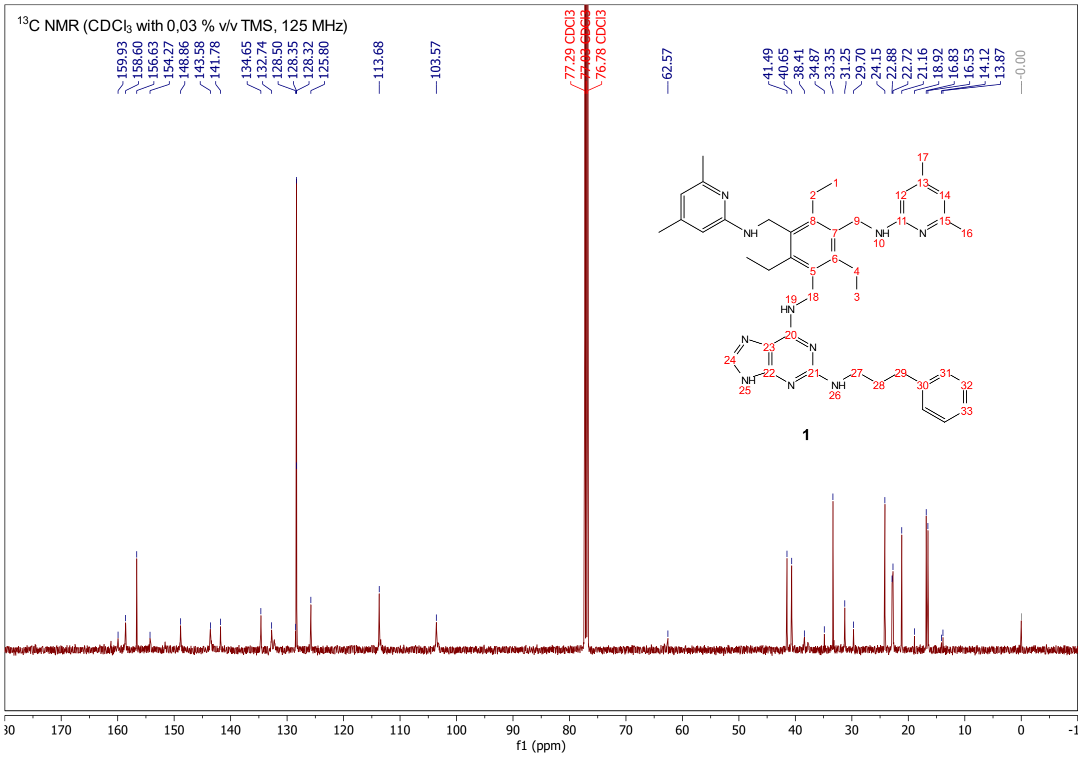

Figure S2: ^{13}C-NMR spectrum of 1-[2-(Phenylpropylamino)-9H-purin-6-yl]aminomethyl-3,5-bis-[(4,6-dimethylpyridin-2-yl)aminomethyl]-2,4,6-triethylbenzene (CHR).

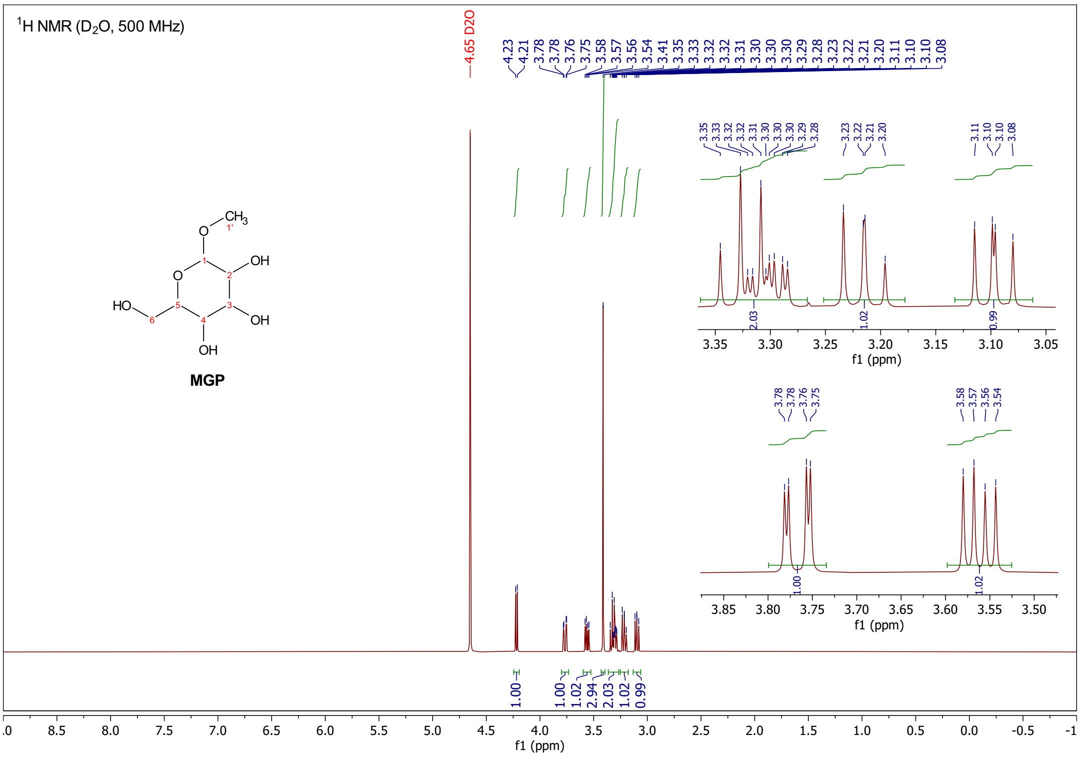

Figure S3: ^{1}H-NMR spectrum of methyl-β-D-glucoside (MGP).

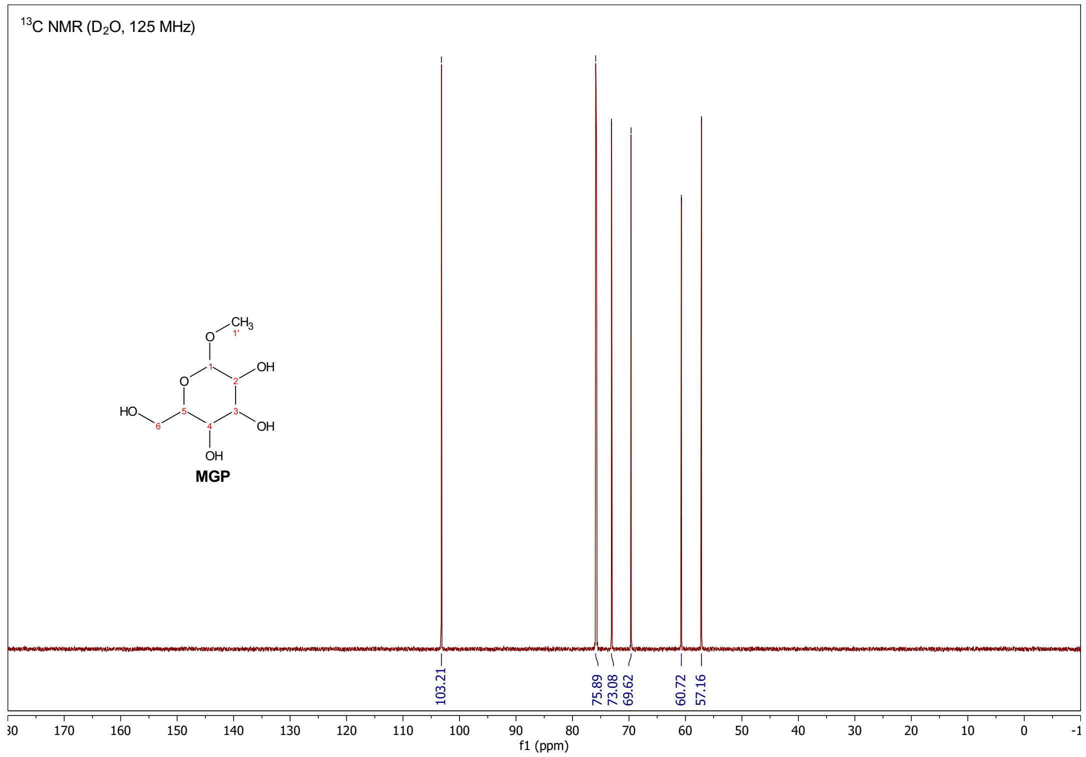

Figure S4: ^{13}C-NMR spectrum of methyl-β-D-glucoside (MGP).

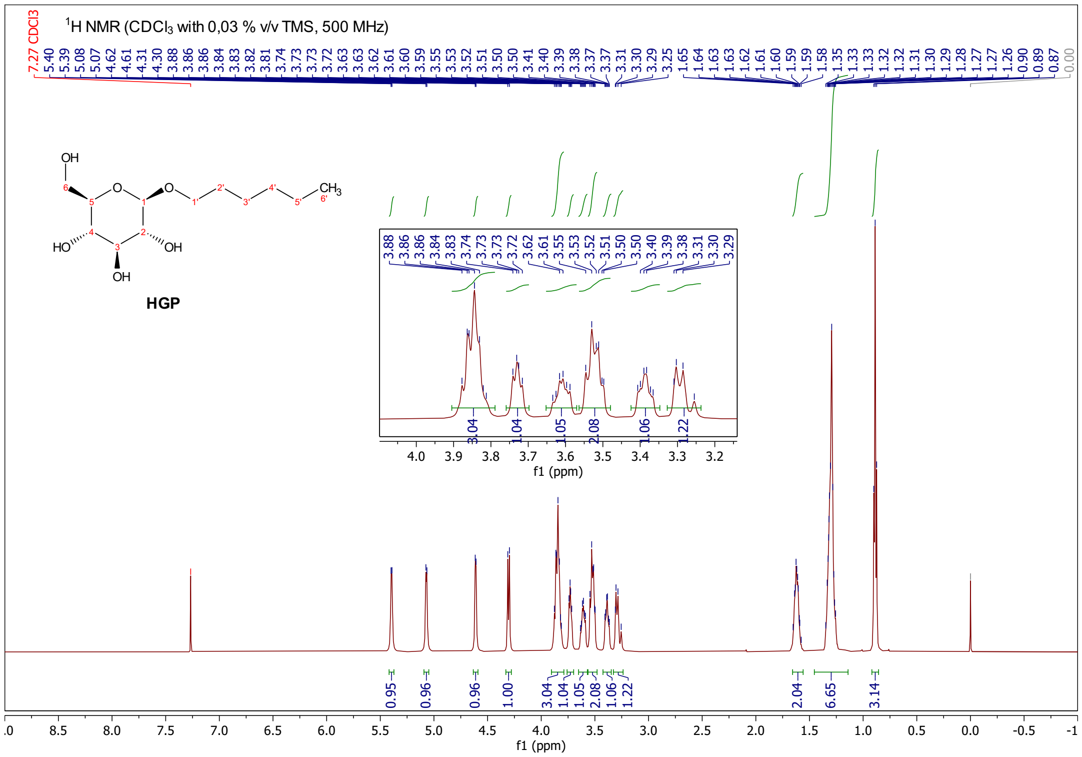

Figure S5: ^{1}H-NMR spectrum of hexyl-β-D-glucoside (HGP).

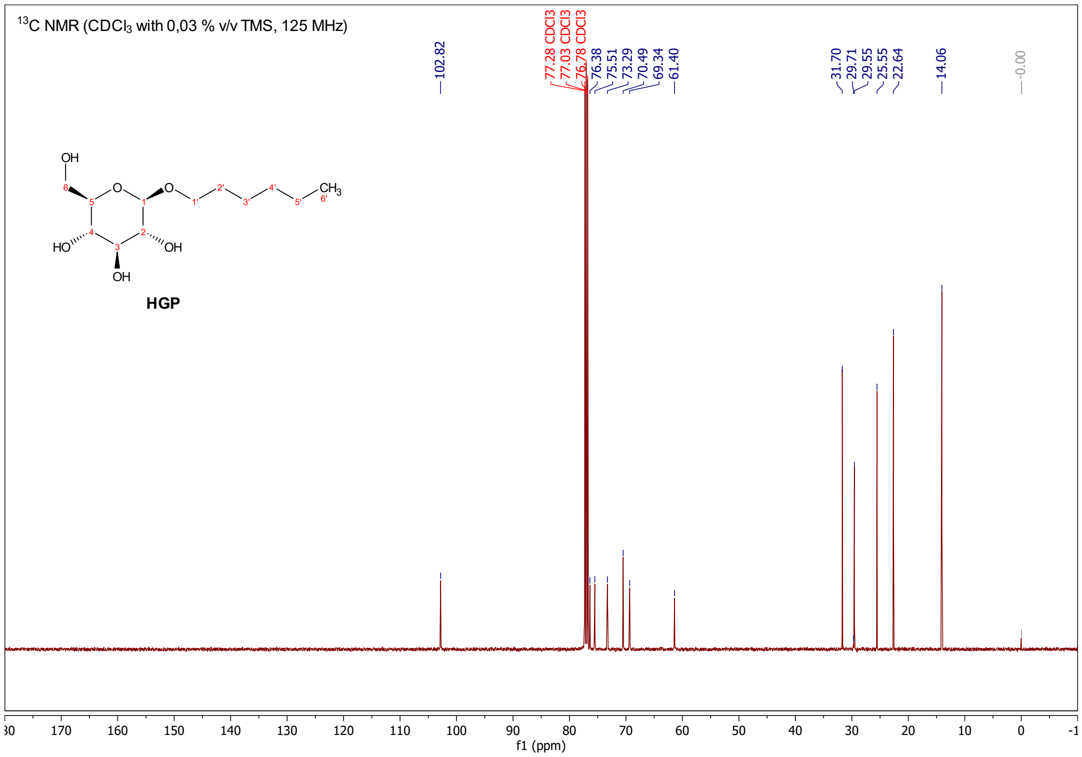

Figure S6: ^{13}C-NMR spectrum of hexyl-β-D-glucoside (MGP).

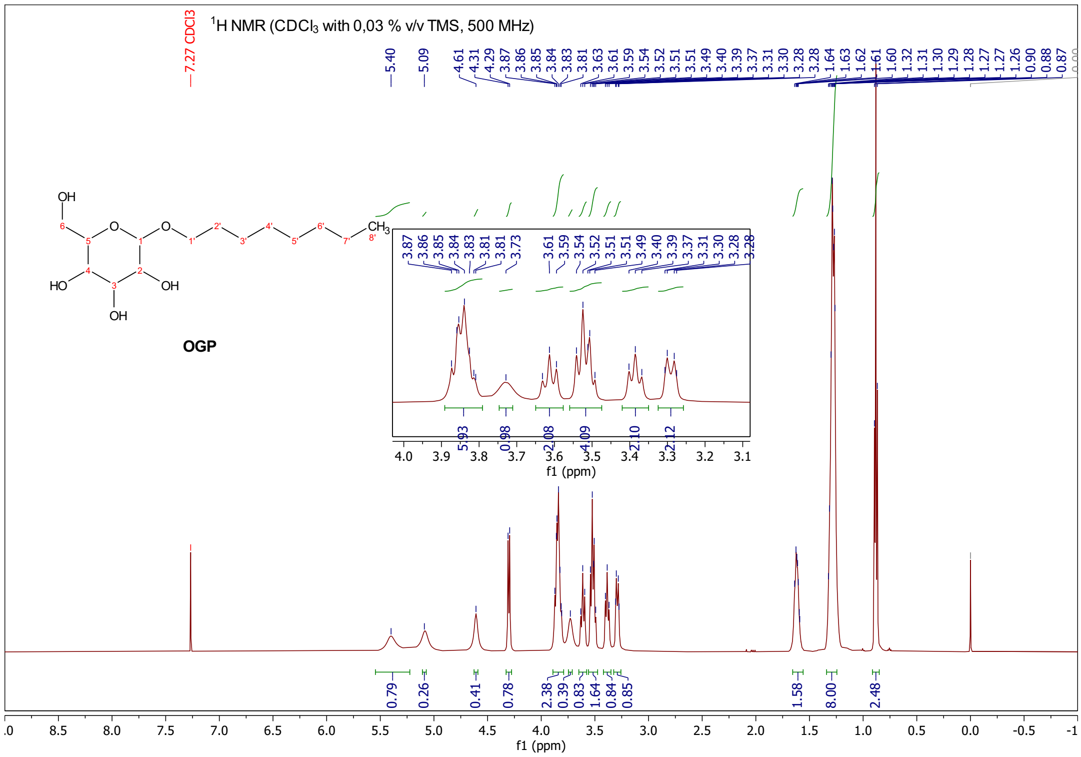

Figure S7: ^{1}H-NMR spectrum of octyl-β-D-glucoside (OGP).

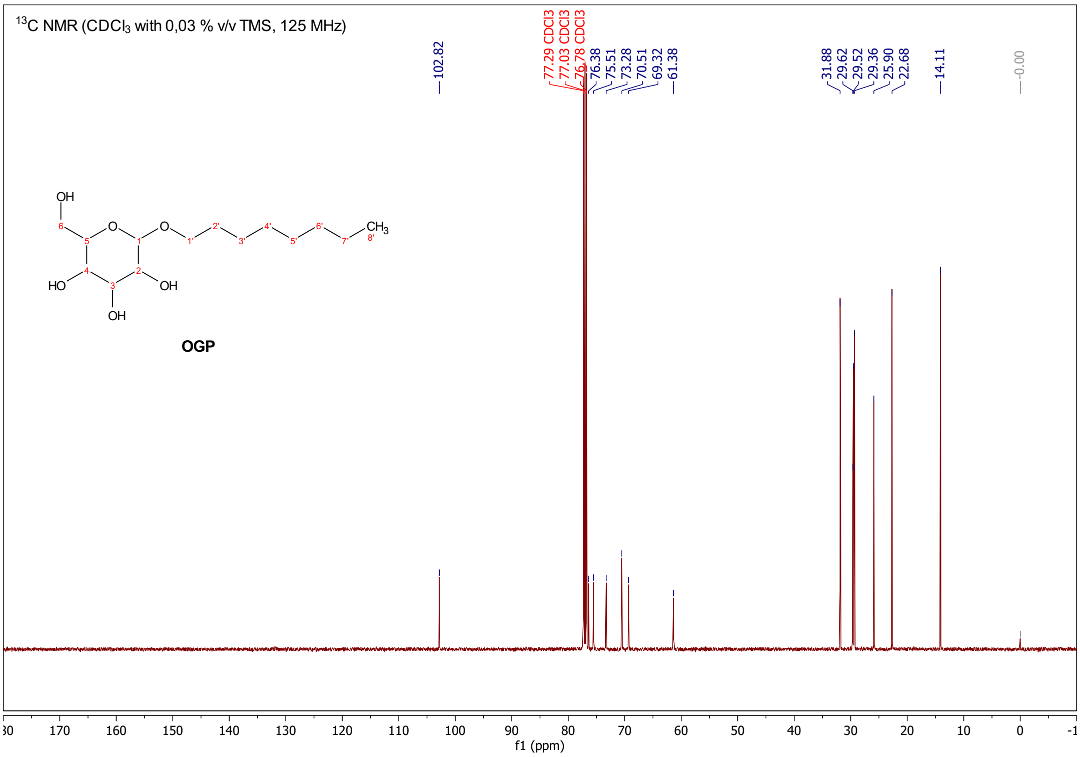

Figure S8: ^{13}C-NMR spectrum of octyl-β-D-glucoside (OGP).

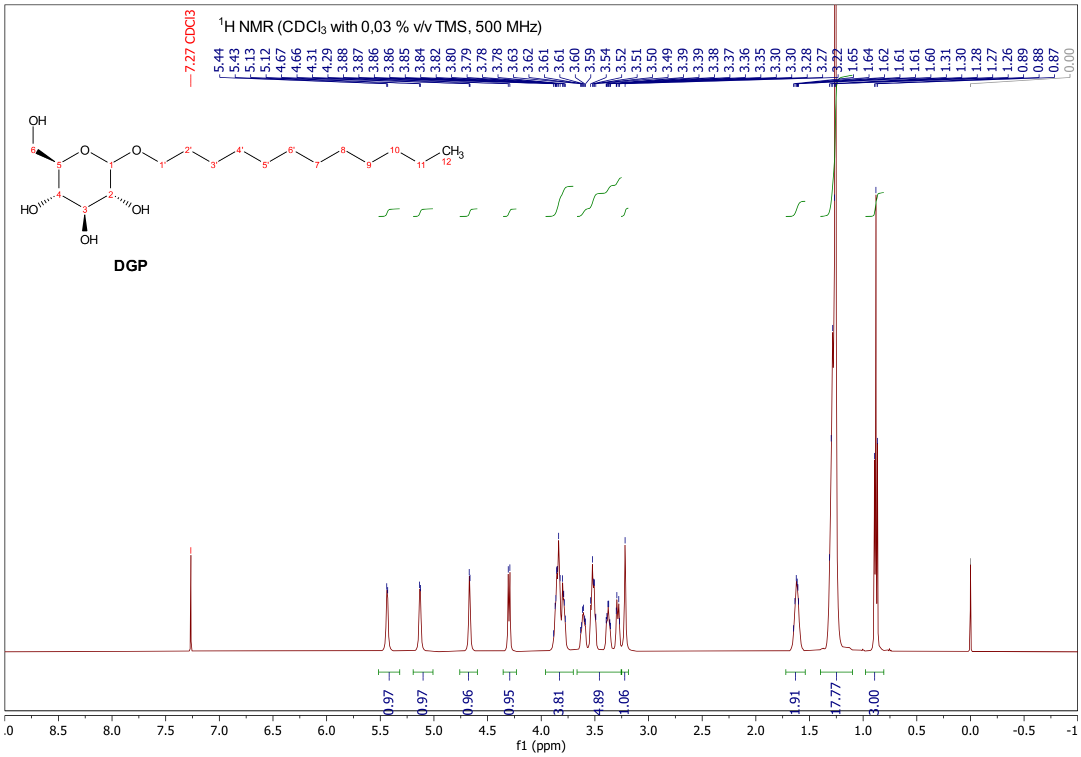

Figure S9: ^{1}H-NMR spectrum of dodecyl-β-D-glucoside (DGP).

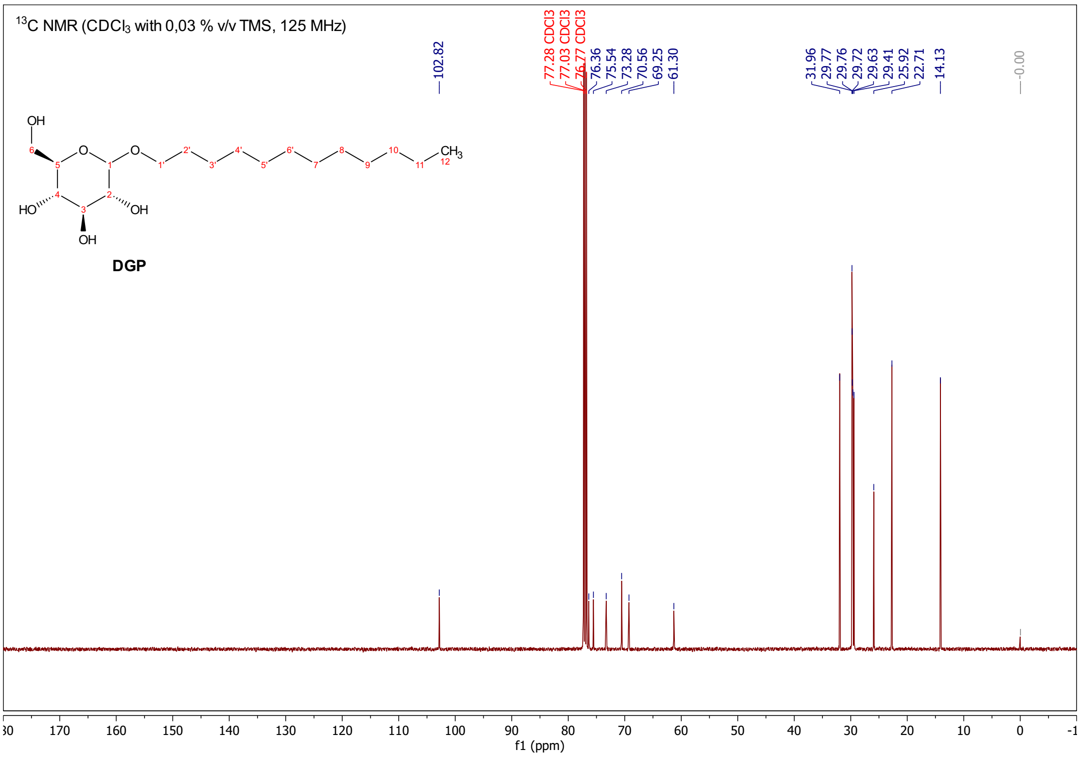

Figure S10: ^{13}C-NMR spectrum of dodecyl-β-D-glucoside (DGP).

S2. Ion Accumulation Time t_{acc} Settings Used for ESI-MS Titration Experiments

Table S1: Ion accumulation time t_{acc} settings for the titration experiments in the positive ion mode depending on the glucoside concentration $[G]$.

	CHR:MGP	CHR:HGP	CHR:OGP	CHR:DGP
$[G]$ / μM	t_{acc} / s	t_{acc} / s	t_{acc} / s	t_{acc} / s
0	0.070	0.055	0.070	0.060
1	0.065	0.070	0.100	0.070
2	0.070	0.070	0.125	0.060
3	0.080	0.070	0.160	0.085
4	0.080	0.065	0.160	0.095
5	0.085	0.060	0.160	0.095
10	0.095	0.050	0.160	0.085
20	0.095	0.040	0.135	0.060
30	0.090	0.035	0.120	0.055
40	0.085	0.025	0.120	0.050
50	0.080	0.020	0.120	0.040
75	0.080	0.025	0.120	0.035
100	0.020	0.020	0.100	0.040
150	0.050	0.020	0.100	0.045
200	0.055	0.015	0.125	0.040

Table S2: Ion accumulation time t_{acc} settings for the titration experiments in the negative ion mode depending on the glucoside concentration $[G]$.

	CHR:MGP	CHR:HGP	CHR:OGP	CHR:DGP
$[G]$ / μM	t_{acc} / s	t_{acc} / s	t_{acc} / s	t_{acc} / s
0	0.023	0.015	0.020	0.020
1	0.028	0.013	0.030	0.025
2	0.035	0.015	0.029	0.025
3	0.024	0.018	0.024	0.025
4	0.022	0.018	0.027	0.025
5	0.018	0.008	0.025	0.040
10	0.018	0.015	0.023	0.080
20	0.022	0.012	0.013	0.060
30	0.022	0.008	0.012	0.050
40	0.022	0.005	0.007	0.050
50	0.023	0.005	0.005	0.050
75	0.020	0.004	0.005	0.050
100	0.020	0.003	0.004	0.040
150	0.015	0.002	0.003	0.040
200	0.015	0.002	0.003	0.040

S3. Derivation of the Expressions for Calculating the Complex Concentration

Here we describe first the general approach for a 1:1 system without assumptions based on the work of Dotsikas et al.[1, 2]. $[H]_0$ and $[G]_0$ are the total concentrations (=initial concentrations) of host/glucoside and guest/receptor. The assignment of host and guest corresponds to an inverse titration. In this case $[H]_0$ is the titration variable and varies troughout the experiment, whereas $[G]_0$ remains constant. Based on the law of conservation of mass and chemical equilibrium, the free host concentration is as follows:

$$[H] = [H]_0 - [HG] = [H]_0 - K[H][G] \tag{1}$$

$$\Rightarrow [H]_0 = [H] + K[H][G] = [H](1 + K[G]) \tag{2}$$

$$\begin{aligned} \Rightarrow [H] &= \frac{[H]_0}{1+K[G]} = \frac{[H]_0}{1+K([G]_0-[HG])} = \frac{[H]_0}{1+K([G]_0-([H]_0-[H]))} \\ &= \frac{[H]_0}{1+K([G]_0-[H]_0+[H])} \end{aligned} \tag{3}$$

$$\begin{aligned} 0 &= [H](1+K([G]_0-[H]_0+[H])) - [H]_0 \\ &= [H] + K[G]_0[H] - K[H][H]_0 + K[H]^2 - [H]_0 \\ &= K[H]^2 + (K[G]_0 - K[H]_0 + 1)[H] - [H]_0 \end{aligned} \tag{4}$$

$$\left(ax^2 + bx + c = 0; \qquad x_{1,2} = \frac{-b \pm \sqrt{b^2-4ac}}{2a} \right)$$

$$[H]_{1,2} = \frac{-(K[G]_0 - K[H]_0 + 1) \pm \sqrt{(K[G]_0 - K[H]_0 + 1)^2 + 4K[H]_0}}{2K} = \frac{A}{2K} \tag{5}$$

Insert Equation 5 into Equation 6 (simple approach[2] from Dotsikas et al. only for $[H] = [H]_0$). The complexation process is expressed with the difference $\Delta I_r = I_r - I_0 \approx I_r$, as the initial complex intensity I_0 is practically zero:

$$I_r \approx \Delta I_r = kK[H]\frac{[G]_0}{1+K[H]} \tag{6}$$

$$\Delta I_r = kK[H]\frac{[G]_0}{1+K[H]} = \frac{\frac{A}{2}[G]_0 k}{1+\frac{A}{2}} = \frac{A[G]_0 k}{2+A} \tag{7}$$

$$= \frac{[G]_0 k \left\{ -(K[G]_0 - K[H]_0 + 1) \pm \sqrt{(K[G]_0 - K[H]_0 + 1)^2 + 4K[H]_0} \right\}}{2 - (K[G]_0 - K[H]_0 + 1) \pm \sqrt{(K[G]_0 - K[H]_0 + 1)^2 + 4K[H]_0}} \tag{8}$$

$$= k[HG] \tag{9}$$

This expression (Equation 9) is similar to the Thordarson approach[3, 4], where the change of the physical property is related to the absolute concentrations (e. g. for NMR experiments). $[H]$, $[G]$ cannot be determined directly, because only initial concentrations are known:

$$K = \frac{[HG]}{[H][G]} = \frac{[HG]}{([H]_0 - [HG])([G]_0 - [HG])} \tag{10}$$

$$\begin{aligned} 0 &= K([H]_0 - [HG])([G]_0 - [HG]) - [HG] \qquad (11)\\ &= K[HG]^2 - [HG](K[H]_0 + K[G]_0 + 1) + K[H]_0[G]_0 \\ &= [HG]^2 - [HG]([H]_0 + [G]_0 + \frac{1}{K}) + [H]_0[G]_0 \end{aligned}$$

$$[HG]_{1,2} = \frac{1}{2}\left([H]_0 + [G]_0 + \frac{1}{K}\right) \pm \sqrt{\frac{1}{4}\left([H]_0 + [G]_0 + \frac{1}{K}\right)^2 - [H]_0[G]_0} \tag{12}$$

We could show that the expressions for $[HG]$ from Dotsikas et al.[1, 2] and Thordarson[3–6] are identical. This makes it possible to simplify the complicated expression Equation 8:

$$\begin{aligned} [HG] &= [HG] \qquad (13)\\ &= \frac{1}{2}\left([H]_0 + [G]_0 + \frac{1}{K}\right) \pm \sqrt{\frac{1}{4}\left([H]_0 + [G]_0 + \frac{1}{K}\right)^2 - [H]_0[G]_0} \qquad (14)\\ &= \frac{[G]_0\left\{-(K[G]_0 - K[H]_0 + 1) \pm \sqrt{(K[G]_0 - K[H]_0 + 1)^2 + 4K[H]_0}\right\}}{2 - (K[G]_0 - K[H]_0 + 1) \pm \sqrt{(K[G]_0 - K[H]_0 + 1)^2 + 4K[H]_0}} \qquad (15) \end{aligned}$$

$$\begin{aligned} &\sqrt{\frac{1}{4}\left([H]_0 + [G]_0 + \frac{1}{K}\right)^2 - [H]_0[G]_0} \qquad (16)\\ &= \sqrt{\frac{1}{4}\left([H]_0^2 + [G]_0^2 + 2[H]_0[G]_0 + \frac{2[H]_0}{K} + \frac{2[G]_0}{K} + \frac{1}{K^2}\right) - [H]_0[G]_0} \\ &= \sqrt{\frac{1}{4}[H]_0^2 + \frac{1}{4}[G]_0^2 + \frac{[H]_0}{2K} + \frac{[G]_0}{2K} + \frac{1}{4K^2} - \frac{1}{2}[H]_0[G]_0} \qquad (17)\\ &= \sqrt{\left(\frac{1}{4}[H]_0^2 + \frac{1}{4}[G]_0^2 + \frac{[H]_0}{2K} + \frac{[G]_0}{2K} + \frac{1}{4K^2} - \frac{1}{2}[H]_0[G]_0\right) \cdot \frac{4K^2}{4K^2}} \\ &= \frac{1}{2K}\sqrt{B} \qquad (18) \end{aligned}$$

$$\sqrt{(K[G]_0 - K[H]_0 + 1)^2 + 4K[H]_0} \tag{19}$$
$$= \sqrt{K^2[G]_0^2 - K^2[G]_0[H]_0 + K[G]_0 - K^2[G]_0[H]_0 + K^2[H]_0^2 - K[H]_0 + K[G]_0 - K[H]_0 + 1 + 4K[H]_0}$$
$$= \sqrt{K^2[G]_0^2 - 2K^2[G]_0[H]_0 + 2K[G]_0 - 2K[H]_0 + K^2[H]_0^2 + 1 + 4K[H]_0}$$
$$= \sqrt{K^2[G]_0^2 - 2K^2[G]_0[H]_0 + 2K[G]_0 + 2K[H]_0 + K^2[H]_0^2 + 1} \tag{20}$$
$$= \sqrt{B} \tag{21}$$

$$\frac{1}{2}\left([H]_0 + [G]_0 + \frac{1}{K}\right) \pm \frac{1}{2K}\sqrt{B} = \frac{[G]_0\left\{-(K[G]_0 - K[H]_0 + 1) \mp \sqrt{B}\right\}}{2 - (K[G]_0 - K[H]_0 + 1) \mp \sqrt{B}} \tag{22}$$
$$= \frac{[G]_0\left\{-(K[G]_0 - K[H]_0 + 1) \mp \sqrt{B}\right\}}{2 - K[G]_0 + K[H]_0 - 1 \mp \sqrt{B}} \tag{23}$$
$$\frac{1}{2}[H]_0 + \frac{1}{2}[G]_0 + \frac{1}{2K} - \frac{1}{2K}\sqrt{B} = \frac{[G]_0\left\{-K[G]_0 + K[H]_0 - 1 + \sqrt{B}\right\}}{1 - K[G]_0 + K[H]_0 + \sqrt{B}} \tag{24}$$

$$\left(\frac{1}{2}[H]_0 + \frac{1}{2}[G]_0 + \frac{1}{2K} - \frac{1}{2K}\sqrt{B}\right)\left(1 - K[G]_0 + K[H]_0 + \sqrt{B}\right) = [G]_0\left(-K[G]_0 + K[H]_0 - 1 + \sqrt{B}\right) \tag{25}$$

$$\left(\frac{1}{2}[H]_0 + \frac{1}{2}[G]_0 + \frac{1}{2K} - \frac{1}{2K}\sqrt{B}\right)\left(1 - K[G]_0 + K[H]_0 + \sqrt{B}\right) \tag{26}$$
$$= \frac{1}{2}[H]_0 - \frac{K}{2}[H]_0[G]_0 + \frac{K}{2}[H]_0^2 + \frac{\sqrt{B}}{2}[H]_0 + \frac{1}{2}[G]_0 - \frac{K}{2}[G]_0^2 + \frac{K}{2}[H]_0[G]_0 + \frac{\sqrt{B}}{2}[G]_0$$
$$+ \frac{1}{2K} - \frac{1}{2}[G]_0 + \frac{1}{2}[H]_0 + \frac{\sqrt{B}}{2K} - \frac{\sqrt{B}}{2K} + \frac{\sqrt{B}}{2}[G]_0 - \frac{\sqrt{B}}{2}[H]_0 - \frac{B}{2K} \tag{27}$$
$$= \frac{1}{2}[H]_0 + \frac{K}{2}[H]_0^2 + \frac{1}{2}[G]_0 - \frac{K}{2}[G]_0^2 + \frac{\sqrt{B}}{2}[G]_0 + \frac{1}{2K} - \frac{1}{2}[G]_0 + \frac{1}{2}[H]_0 + \frac{\sqrt{B}}{2}[G]_0 - \frac{B}{2K} \tag{28}$$
$$= [H]_0 + \frac{K}{2}[H]_0^2 - \frac{K}{2}[G]_0^2 + \sqrt{B}[G]_0 + \frac{1}{2K} - \frac{B}{2K} \tag{29}$$
$$= -K[G]_0^2 + K[G]_0[H]_0 - [G]_0 + [G]_0\sqrt{B} \tag{30}$$

$$\Leftrightarrow [H]_0 + \frac{K}{2}[H]_0^2 + \frac{K}{2}[G]_0^2 + \frac{1}{2K} - \frac{B}{2K} = K[G]_0[H]_0 - [G]_0 \tag{31}$$

$$\Leftrightarrow [H]_0 + \frac{K}{2}[H]_0^2 + \frac{K}{2}[G]_0^2 + \frac{1}{2K} - \frac{K}{2}[G]_0^2 + K[G]_0[H]_0 - [G]_0 - [H]_0 - \frac{K}{2}[H]_0^2 - \frac{1}{2K} \tag{32}$$

$$= K[G]_0[H]_0 - [G]_0 \tag{33}$$

$$0 = 0 \qquad \text{q. e. d.} \tag{34}$$

It can be shown that the equality also exists if the signs of the root terms are inverted.

$$\Delta I_r = k\left(\frac{1}{2}\left([H]_0 + [G]_0 + \frac{1}{K}\right) \pm \sqrt{\frac{1}{4}\left([H]_0 + [G]_0 + \frac{1}{K}\right)^2 - [H]_0[G]_0}\right) \tag{35}$$

ΔI_r, $[G]_0$ and $[H]_0$ are known, k and K are variables. These parameters can be determined using an iterative solution method, whereby one solution must be discarded in each case (either trivial or negative). Determined k values for triplicate analyses are shown in Table S3.

Table S3: The k values from the linear and nonlinear fitting approaches of experimental titration data. The unit of k is 1/µmol.

	linear approach			nonlinear approach		
complex (ion mode)	k_1	k_2	k_3	k_1	k_2	k_3
CHR:MGP(+)	0.0582	0.0557	0.0515	0.0797	0.0775	0.0785
CHR:MGP(-)	0.0080	0.0113	0.0102	0.0153	0.0140	0.0142
CHR:HGP(+)	0.1289	0.1151	0.1221	0.1103	0.1100	0.1099
CHR:HGP(-)	0.0577	0.0580	0.0756	0.0682	0.0647	0.0650
CHR:OGP(+)	0.1209	0.1165	0.1209	0.1274	0.1300	0.1288
CHR:OGP(-)	0.0880	0.0919	0.0963	0.0910	0.0906	0.0936
CHR:DGP(+)	0.1562	0.1451	0.1459	0.1259	0.1261	0.1253
CHR:DGP(-)	0.0790	0.0801	0.0794	0.0831	0.0876	0.0824

S4. Development and Optimization of Analysis Routines for ESI(+/-)-MS

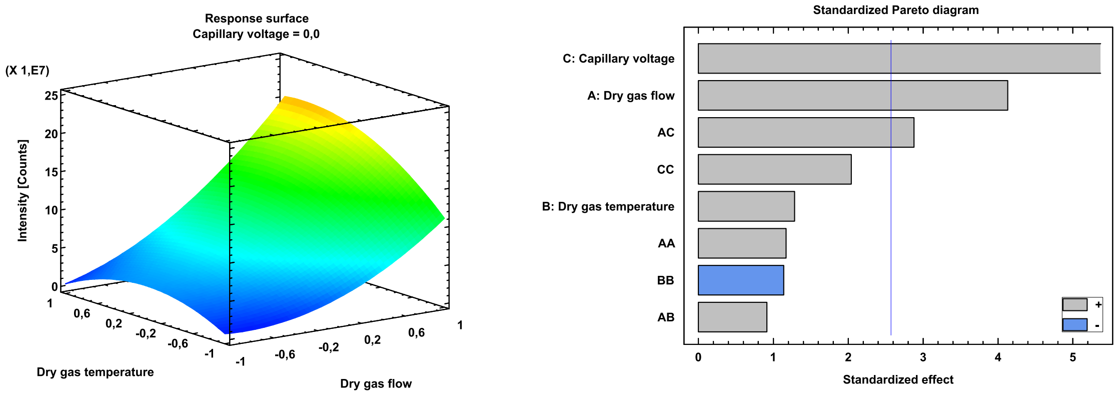

Figure S11: The Box-Behnken plan (three factors) was used to optimize the ESI parameters for methanol usage, resulting in a response surface. The complex ion abundances are presented in a color-coded format, with green/blue indicating low intensity, yellow indicating medium intensity, and red indicating high intensity. Additionally, a standardized Pareto diagram was generated as a result of the optimization process. The blue vertical line in the diagram represents the level of significance (5 %).

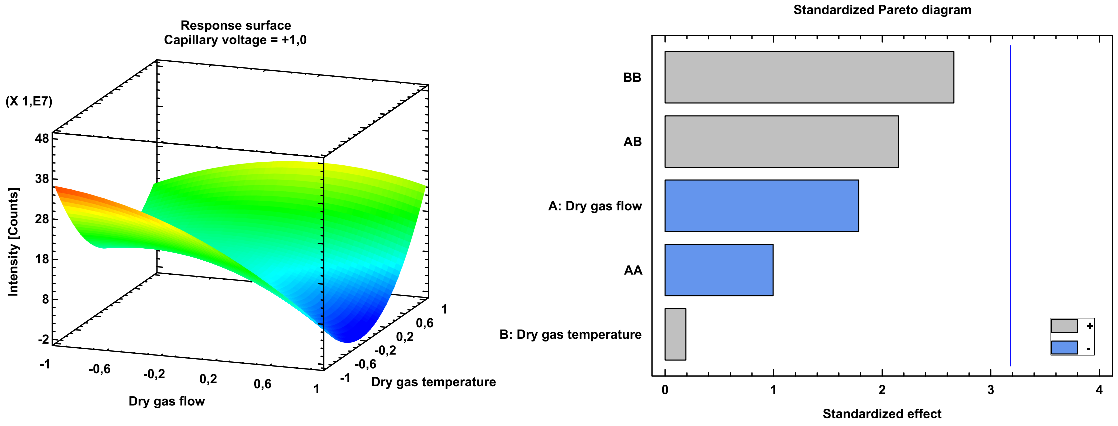

Figure S12: In the second step, a 3^2 factorial design was used to further optimize the ESI parameters for methanol usage, resulting in a response surface. The complex ion abundances are presented in a color-coded format, with green/blue indicating low intensity, yellow indicating medium intensity, and red indicating high intensity. Additionally, a standardized Pareto diagram was generated as a result of the optimization process. The blue vertical line in the diagram represents the level of significance (5 %).

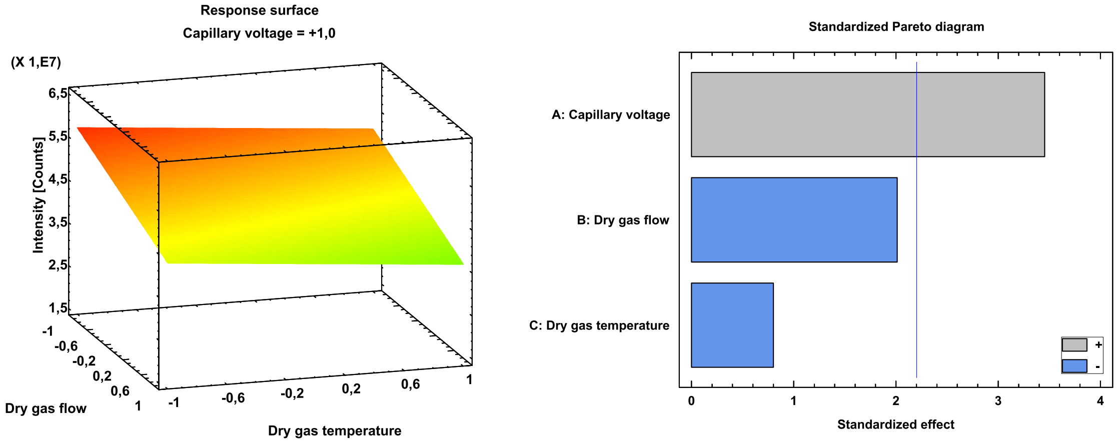

Figure S13: The Box-Behnken plan (three factors) was used to optimize the ESI parameters for acetone usage, resulting in a response surface. The complex ion abundances are presented in a color-coded format, with green/blue indicating low intensity, yellow indicating medium intensity, and red indicating high intensity. Additionally, a standardized Pareto diagram was generated as a result of the optimization process. The blue vertical line in the diagram represents the level of significance (5 %).

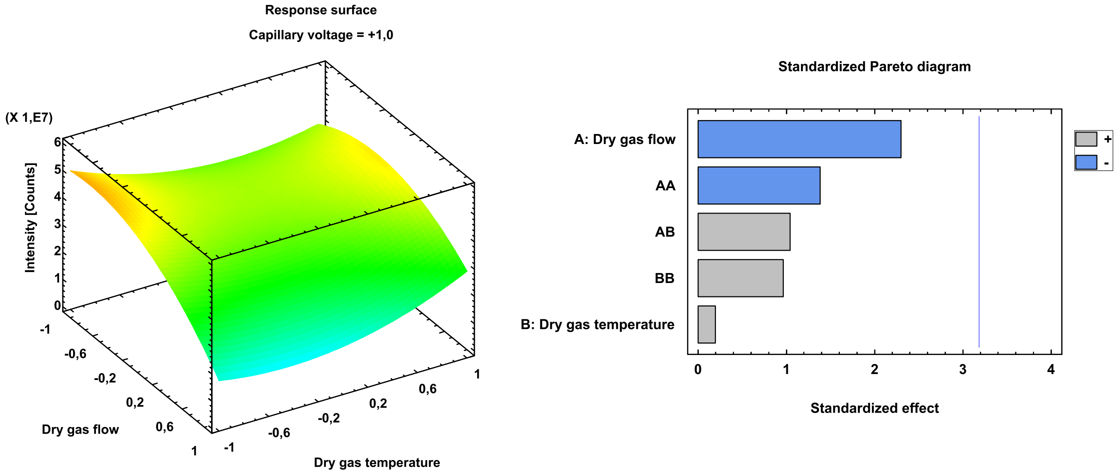

Figure S14: In the second step, a 3^2 factorial design was used to further optimize the ESI parameters for acetone usage, resulting in a response surface. The complex ion abundances are presented in a color-coded format, with green/blue indicating low intensity, yellow indicating medium intensity, and red indicating high intensity. Additionally, a standardized Pareto diagram was generated as a result of the optimization process. The blue vertical line in the diagram represents the level of significance (5 %).

S5. Mass spectrometrical results

Table S4: Most abundant molecular ions from ESI(+) and ESI(-) mass spectra of the equimolar solutions for CHR:MGP as well as CHR:HGP complex systems with their respective masses, assigned ion formulas, absolute ion intensities and S/N. Ion masses were calculated with Bruker Compass IsotopePattern tool and used for calibration lists and further data processing (GP - glucopyranoside).

MGP	molecular ion	m/z	I	S/N
$[GP + Na]^+$	217.068259	$C_7H_{14}O_6Na$	7901945	63.4
$[CHR + H_2]^{2+}$	356.233922	$C_{43}H_{56}N_{10}$	6765893	57.8
$[(GP)_2 + Na]^+$	411.147297	$C_{14}H_{28}O_{12}Na$	28837104	247.2
$[CHR + H]^+$	711.460583	$C_{43}H_{55}N_{10}$	410782272	3123.3
$[CHR + Na]^+$	733.442535	$C_{43}H_{54}N_{10}Na$	89134160	665.6
$[CHR{:}GP + H]^+$	905.539606	$C_{50}H_{69}O_6N_{10}$	12330000	83.4
$[CHR{:}GP + Na]^+$	927.521551	$C_{50}H_{68}O_6N_{10}Na$	2858777	17.3
$[(CHR)_2 + H]^+$	1421.914012	$C_{86}H_{109}N_{20}$	73765216	390.9
$[(CHR)_2 + Na]^+$	1443.895775	$C_{86}H_{108}N_{20}Na$	6102953	30.5
$[(CHR)_2{:}GP + H]^+$	1615.992898	$C_{93}H_{123}N_{20}O_6$	1595459	5.7
$[(CHR)_2{:}GP + Na]^+$	1637.974842	$C_{93}H_{122}N_{20}O_6Na$	245244	5.3
$[GP - H]^-$	193.071762	$C_7H_{13}O_6$	2106425	15.2
$[GP + Na - 2\,H]^-$	215.053706	$C_7H_{12}O_6Na$	891293	5.3
$[(GP)_2 - H]^-$	387.150800	$C_{14}H_{27}O_{12}$	10623463	90.2
$[(GP)_2 + Na - 2\,H]^-$	409.132744	$C_{14}H_{26}O_{12}Na$	1523678	11.1
$[CHR - H]^-$	709.446015	$C_{43}H_{53}N_{10}$	6407484	46.6
$[CHR + Na - 2\,H]^-$	731.427960	$C_{43}H_{52}N_{10}Na$	7368909	53.0
$[CHR{:}GP - H]^-$	903.525054	$C_{50}H_{67}O_6N_{10}$	6037426	39.8
$[CHR{:}GP + Na - 2\,H]^-$	925.506998	$C_{50}H_{66}O_6N_{10}Na$	1113817	5.6
$[(CHR)_2 - H]^-$	1419.899307	$C_{86}H_{107}N_{20}$	6458509	32.2
$[(CHR)_2{:}GP - H]^-$	1613.978345	$C_{93}H_{121}N_{20}O_6$	1755462	6.4
$[(CHR)_2{:}GP + Na - 2\,H]^-$	1635.960290	$C_{93}H_{120}N_{20}O_6Na$	1671644	5.9
HGP				
$[GP + Na]^+$	287.146509	$C_{12}H_{24}O_6Na$	88016592	759.2
$[CHR + H_2]^{2+}$	356.233922	$C_{43}H_{56}N_{10}$	2708926	21.9
$[(GP)_2 + Na]^+$	551.303798	$C_{24}H_{48}O_{12}Na$	70961488	572.6
$[CHR + H]^+$	711.460583	$C_{43}H_{55}N_{10}$	262933856	1988.6
$[CHR + Na]^+$	733.442535	$C_{43}H_{54}N_{10}Na$	92280752	687.8
$[CHR{:}GP + H]^+$	975.617857	$C_{55}H_{79}N_{10}O_6$	89557880	584.7
$[CHR{:}GP + Na]^+$	997.599801	$C_{55}H_{78}N_{10}O_6Na$	23533076	152.2
$[(CHR)_2 + H]^+$	1421.914012	$C_{86}H_{109}N_{20}$	54235852	285.8

$[(CHR)_2 + Na]^+$	1443.895775	$C_{86}H_{108}N_{20}Na$	7462749	37.6
$[(CHR)_2{:}GP + H]^+$	1686.071149	$C_{98}H_{133}N_{20}O_6$	1514131	5.2
$[(CHR)_2{:}GP + Na]^+$	1708.053093	$C_{98}H_{132}N_{20}O_6Na$	1583058	5.5
$[GP - H]^-$	263.150012	$C_{12}H_{23}O_6$	19644140	164.3
$[GP + Na - 2\,H]^-$	285.131956	$C_{12}H_{22}O_6Na$	2429128	18.9
$[(GP)_2 - H]^-$	527.307301	$C_{24}H_{47}O_{12}$	84919064	693.2
$[(GP)_2 + Na - 2\,H]^-$	549.289245	$C_{24}H_{46}O_{12}Na$	10700876	84.4
$[CHR - H]^-$	709.446015	$C_{43}H_{53}N_{10}$	635702720	4812.1
$[CHR + Na - 2\,H]^-$	731.427960	$C_{43}H_{52}N_{10}Na$	6142045	43.9
$[CHR{:}GP - H]^-$	973.603304	$C_{55}H_{77}N_{10}O_6$	39555400	256.2
$[CHR{:}GP + Na - 2\,H]^-$	995.585248	$C_{55}H_{76}N_{10}O_6Na$	1113217	5.3
$[(CHR)_2 - H]^-$	1419.899307	$C_{86}H_{107}N_{20}$	15045158	77.6
$[(CHR)_2{:}GP - H]^-$	1684.056596	$C_{98}H_{131}N_{20}O_6$	1522008	5.2
$[(CHR)_2{:}GP + Na - 2\,H]^-$	1706.038540	$C_{98}H_{130}N_{20}O_6Na$	1476466	5.0

Table S5: Most abundant molecular ions from ESI(+) and ESI(-) mass spectra of the equimolar solutions for CHR:OGP as well as CHR:DGP complex systems with their respective masses, assigned ion formulas, absolute ion intensities and S/N. Ion masses were calculated with Bruker Compass IsotopePattern tool and used for calibration lists and further data processing (GP - glucopyranoside).

OGP molecular ion	m/z	ion formula	I	S/N
$[GP + Na]^+$	315.177808	$C_{14}H_{28}O_6Na$	92271912	809.9
$[CHR + H_2]^{2+}$	356.233922	$C_{43}H_{56}N_{10}$	2672131	21.5
$[(GP)_2 + Na]^+$	607.366441	$C_{28}H_{56}O_{12}Na$	28171044	221.1
$[CHR + H]^+$	711.460583	$C_{43}H_{55}N_{10}$	290197248	2187.0
$[CHR + Na]^+$	733.442535	$C_{43}H_{54}N_{10}Na$	81533464	603.9
$[CHR{:}GP + H]^+$	1003.649129	$C_{57}H_{83}N_{10}O_6$	104112536	675.3
$[CHR{:}GP + Na]^+$	1025.631060	$C_{57}H_{82}N_{10}O_6Na$	16330069	100.8
$[(CHR)_2 + H]^+$	1421.914012	$C_{86}H_{109}N_{20}$	43948564	231.0
$[(CHR)_2 + Na]^+$	1443.895775	$C_{86}H_{108}N_{20}Na$	6449267	32.2
$[(CHR)_2{:}GP + H]^+$	1714.102449	$C_{100}H_{137}N_{20}O_6$	1500584	5.1
$[(CHR)_2{:}GP + Na]^+$	1736.084393	$C_{100}H_{136}N_{20}O_6Na$	1481949	5.0
$[GP - H]^-$	291.181312	$C_{14}H_{27}O_6$	73108536	626.6
$[GP + Na - 2\,H]^-$	313.163256	$C_{14}H_{26}O_6Na$	3963383	32.7
$[(GP)_2 - H]^-$	583.369901	$C_{28}H_{55}O_{12}$	198693616	1587.9
$[(GP)_2 + Na - 2\,H]^-$	605.351845	$C_{28}H_{54}O_{12}Na$	13900296	108.3
$[CHR - H]^-$	709.446015	$C_{43}H_{53}N_{10}$	487851456	3703.9
$[CHR + Na - 2\,H]^-$	731.427960	$C_{43}H_{52}N_{10}Na$	6115174	43.8
$[CHR{:}GP - H]^-$	1001.634604	$C_{57}H_{81}N_{10}O_6$	77109288	505.1

$[CHR{:}GP + Na - 2\,H]^-$	1023.616548	$C_{57}H_{80}N_{10}O_6Na$	3268887	18.9
$[(CHR)_2 - H]^-$	1419.899307	$C_{86}H_{107}N_{20}$	31622376	165.5
$[(CHR)_2{:}GP - H]^-$	1712.087896	$C_{100}H_{135}N_{20}O_6$	1558747	5.4
$[(CHR)_2{:}GP + Na - 2\,H]^-$	1734.069840	$C_{100}H_{134}N_{20}O_6Na$	1586031	5.5
DGP				
$[GP + Na]^+$	371.240410	$C_{18}H_{36}O_6Na$	96956184	848.7
$[CHR + H_2]^{2+}$	356.233922	$C_{43}H_{56}N_{10}$	3365650	27.8
$[(GP)_2 + Na]^+$	719.491598	$C_{36}H_{72}O_{12}Na$	35983352	267.2
$[CHR + H]^+$	711.460583	$C_{43}H_{55}N_{10}$	378276896	2868.4
$[CHR + Na]^+$	733.442535	$C_{43}H_{54}N_{10}Na$	64231060	478.5
$[CHR{:}GP + H]^+$	1059.711757	$C_{61}H_{91}N_{10}O_6$	96254920	605.4
$[CHR{:}GP + Na]^+$	1081.693701	$C_{61}H_{90}N_{10}O_6Na$	20073056	124.7
$[(CHR)_2 + H]^+$	1421.914012	$C_{86}H_{109}N_{20}$	59612416	312.6
$[(CHR)_2 + Na]^+$	1443.895775	$C_{86}H_{108}N_{20}Na$	5418089	26.6
$[(CHR)_2{:}GP + H]^+$	1770.165049	$C_{104}H_{145}N_{20}O_6$	3058839	12.1
$[(CHR)_2{:}GP + Na]^+$	1792.146993	$C_{104}H_{144}N_{20}O_6Na$	2469163	9.4
$[GP - H]^-$	347.243912	$C_{18}H_{35}O_6$	78147680	686.6
$[GP + Na - 2\,H]^-$	369.225857	$C_{18}H_{34}O_6Na$	5112828	42.6
$[(GP)_2 - H]^-$	695.495101	$C_{36}H_{71}O_{12}$	95291064	717.3
$[(GP)_2 + Na - 2\,H]^-$	717.477046	$C_{36}H_{70}O_{12}Na$	11882917	86.5
$[CHR - H]^-$	709.446015	$C_{43}H_{53}N_{10}$	317459904	2394.3
$[CHR + Na - 2\,H]^-$	731.427960	$C_{43}H_{52}N_{10}Na$	7256151	52.1
$[CHR{:}GP - H]^-$	1057.697204	$C_{61}H_{89}N_{10}O_6$	63226140	397.3
$[CHR{:}GP + Na - 2\,H]^-$	1079.679148	$C_{61}H_{88}N_{10}O_6Na$	4304646	25.2
$[(CHR)_2 - H]^-$	1419.899307	$C_{86}H_{107}N_{20}$	13276497	68.2
$[(CHR)_2{:}GP - H]^-$	1768.150496	$C_{104}H_{143}N_{20}O_6$	1570147	5.4
$[(CHR)_2{:}GP + Na - 2\,H]^-$	1790.132440	$C_{104}H_{142}N_{20}O_6Na$	1552194	5.3

S6. Further Results from ESI-MS Titration Experiments and Determination of Dissociation Constants

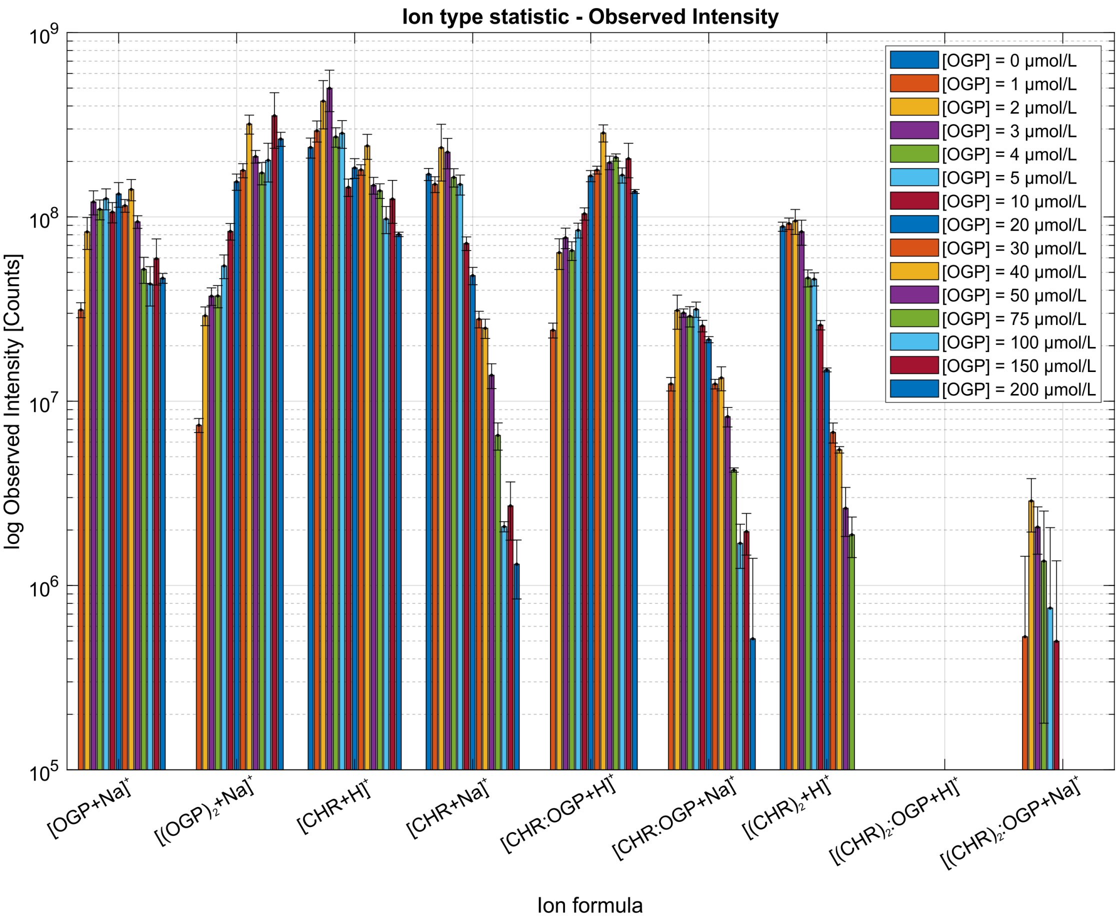

Figure S15: Statistical plot of different ion types with their absolute intensities (error bars represent the standard deviation for triplicate analyses) as a function of the sugar concentration in the range 0 – 200 µmol/L, which dominate the mass spectra recorded during ESI titration in positive mode. As the concentration of OGP increases from sample to sample, the intensity of the singly protonated complex also increases, while the intensity of the receptor decreases. This decrease in intensity can also be observed for the sodium adduct of the receptor and the singly protonated dimer. In line with theoretical expectations, the abundance of the sodium adduct of the sugar and its dimer also increases with increasing OGP concentration, although not to the same extent, as the ions differ in size and the associated ionization efficiency. The sodium adduct of the complex also becomes increasingly less abundant with increasing sugar concentration, as the sodium ions tend to form adducts with the dimer of the sugar. For the 2:1 complex, peaks can only be observed up to a concentration of 10 µmol/L, sometimes only in certain individual analyses.

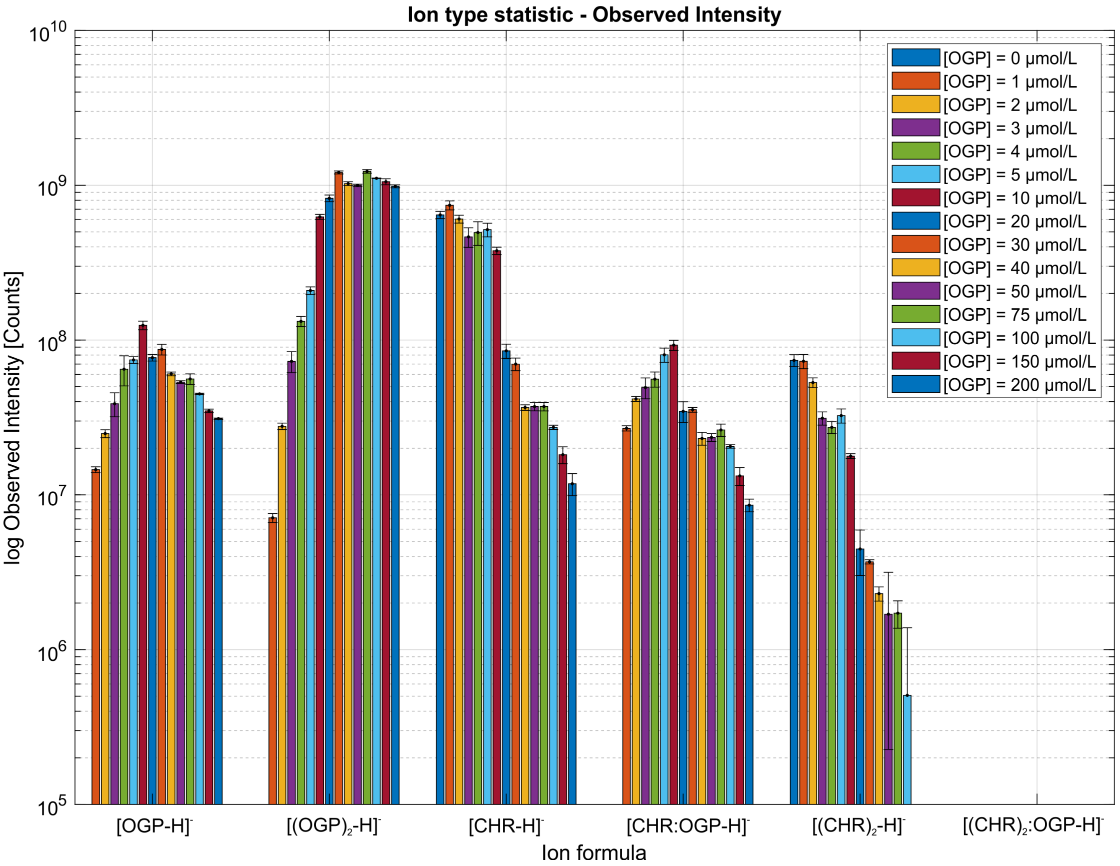

Figure S16: Statistical plot of different singly deprotonated ion types with their absolute intensities (error bars represent the standard deviation for triplicate analyses) as a function of the sugar concentration in the range 0 – 200 µmol/L, which dominate the mass spectra recorded during ESI titration in negative mode. As the OGP concentration increases from sample to sample, a permanent increase in intensity can only be observed for the OGP dimer. For high concentrations above 10 µmol/L (CHR:OGP ratio greater than 2:1), a saturation appears to have been reached. For the singly deprotonated OGP ion and the complex, an approximately equal increase can also be observed initially, but the intensities drop again from the concentration mentioned. The decreasing abundance for the receptor and its dimer corresponds to theoretical expectations. The 2:1 complex could not be observed.

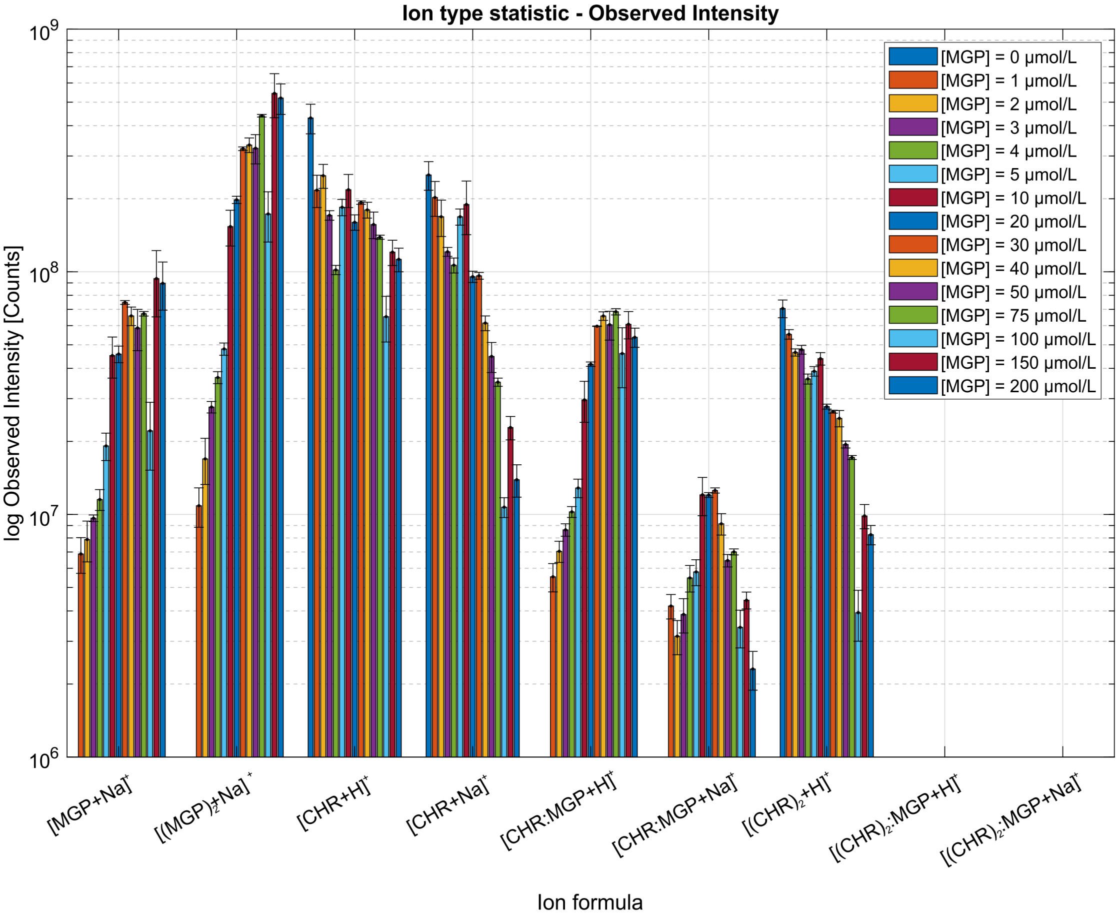

Figure S17: Statistical plot of different ion types with their absolute intensities (error bars represent the standard deviation for triplicate analyses) as a function of the MGP concentration in the range 0 – 200 µmol/L, which dominate the mass spectra recorded during ESI titration in positive mode.

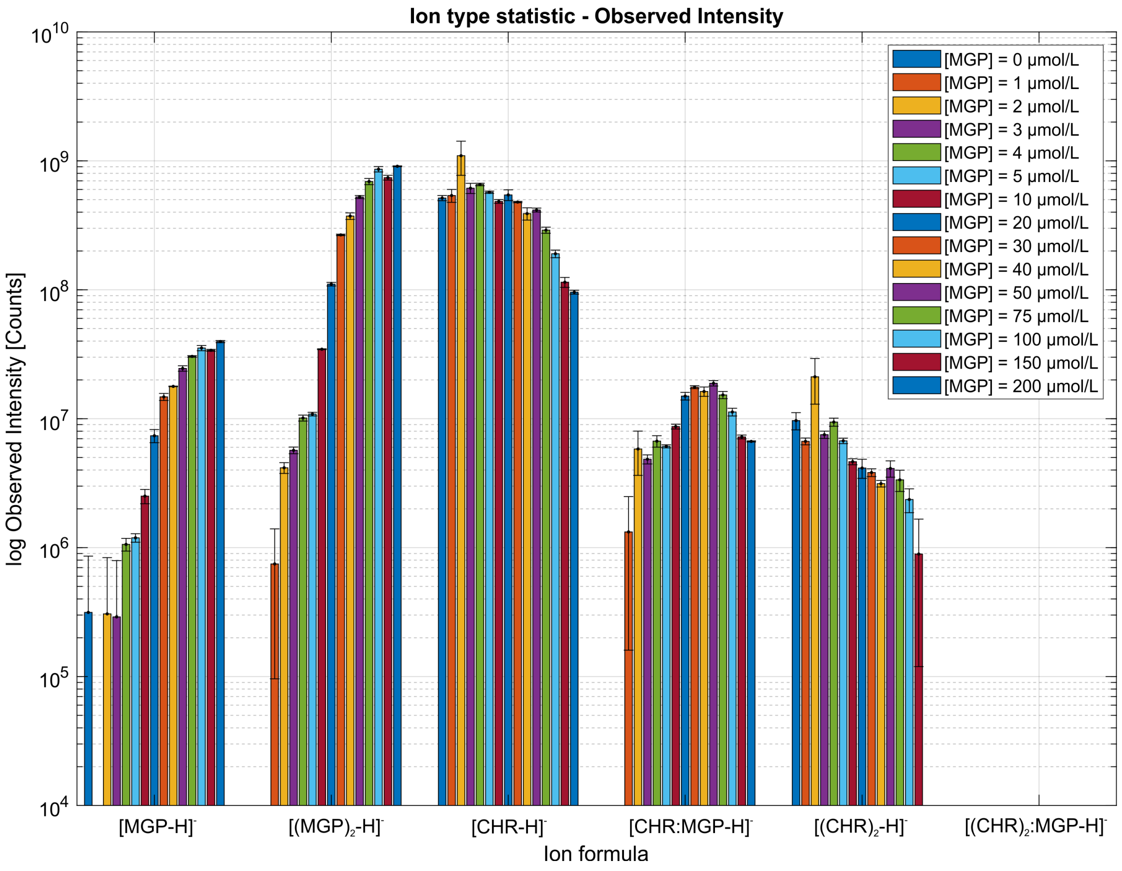

Figure S18: Statistical plot of different singly deprotonated ion types with their absolute intensities (error bars represent the standard deviation for triplicate analyses) as a function of the MGP concentration in the range 0 – 200 µmol/L, which dominate the mass spectra recorded during ESI titration in negative mode.

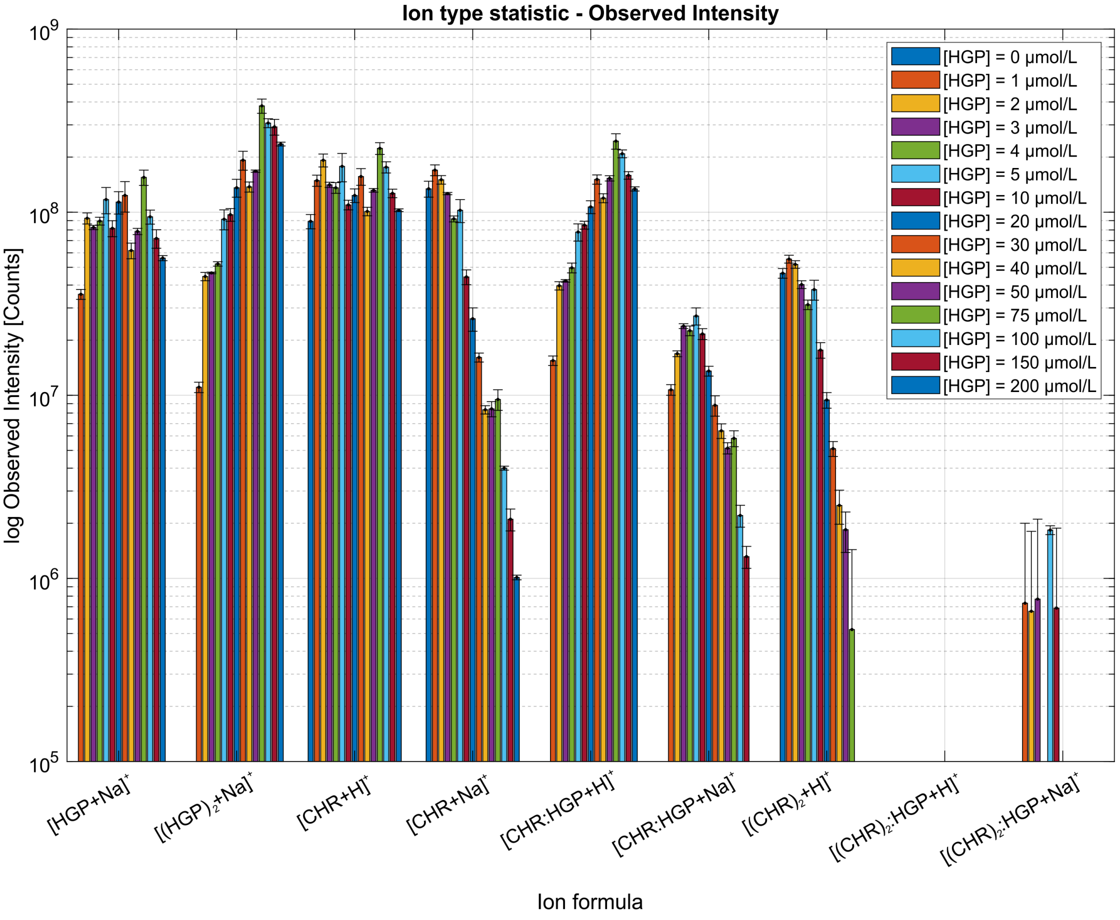

Figure S19: Statistical plot of different ion types with their absolute intensities (error bars represent the standard deviation for triplicate analyses) as a function of the HGP concentration in the range 0 – 200 µmol/L, which dominate the mass spectra recorded during ESI titration in positive mode.

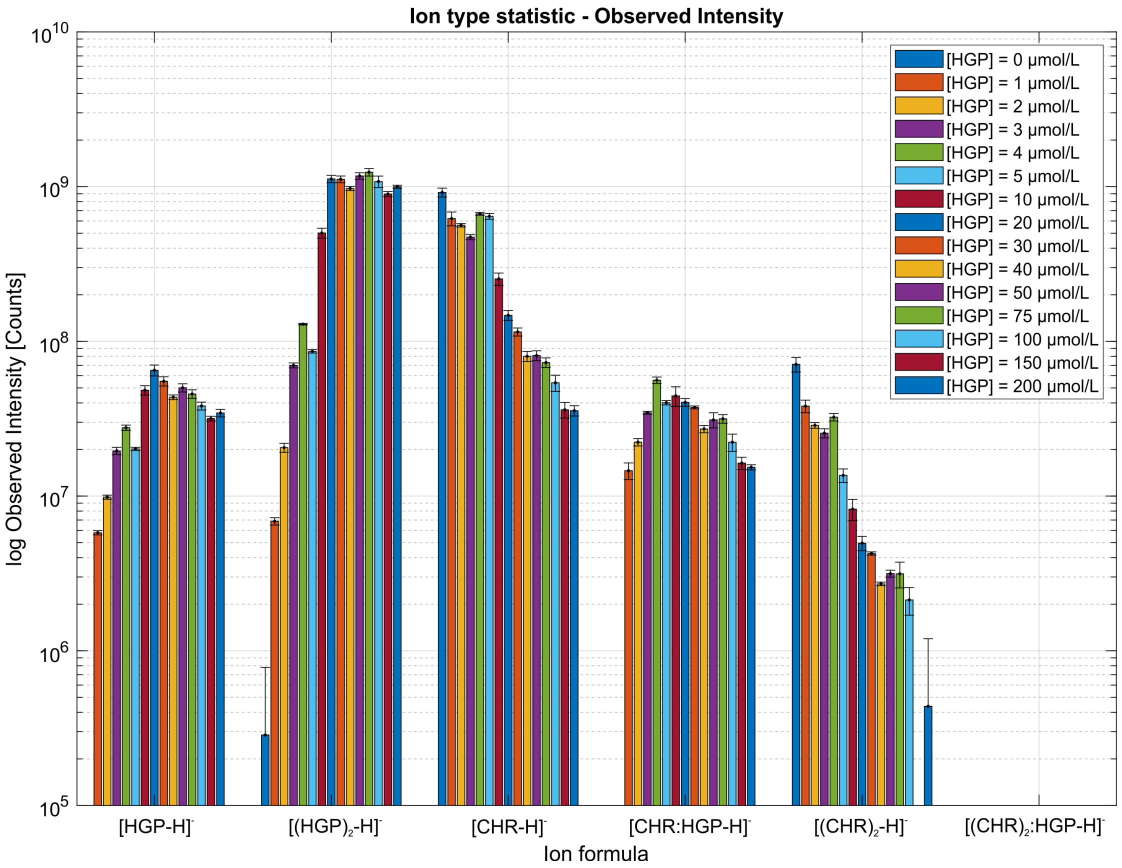

Figure S20: Statistical plot of different singly deprotonated ion types with their absolute intensities (error bars represent the standard deviation for triplicate analyses) as a function of the HGP concentration in the range 0 – 200 µmol/L, which dominate the mass spectra recorded during ESI titration in negative mode.

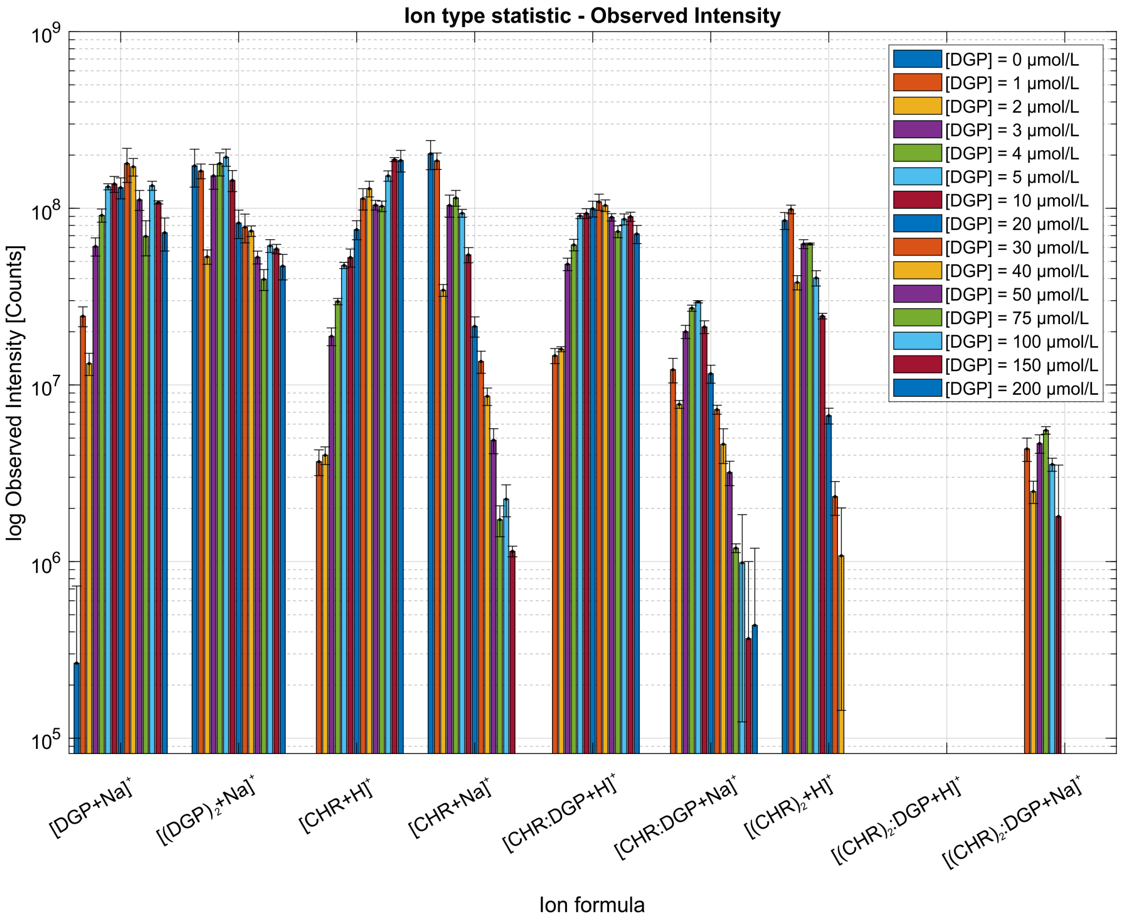

Figure S21: Statistical plot of different ion types with their absolute intensities (error bars represent the standard deviation for triplicate analyses) as a function of the DGP concentration in the range 0 – 200 µmol/L, which dominate the mass spectra recorded during ESI titration in positive mode.

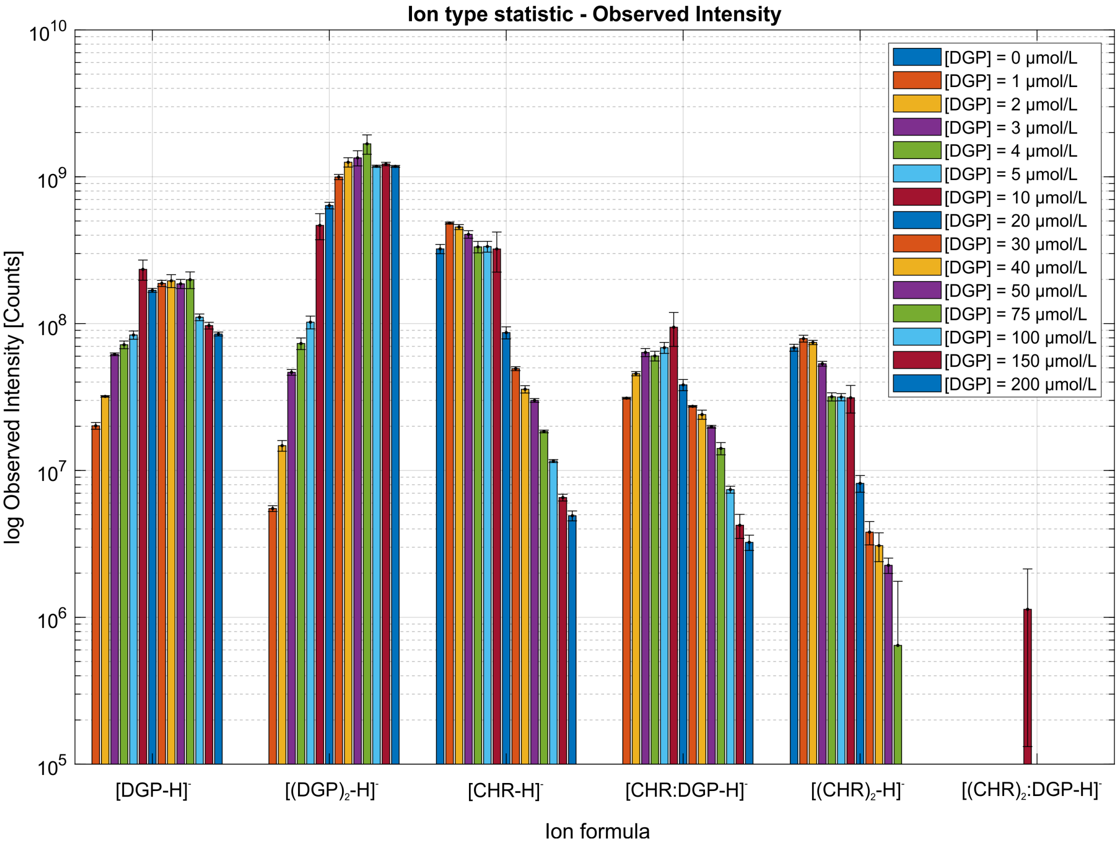

Figure S22: Statistical plot of different singly deprotonated ion types with their absolute intensities (error bars represent the standard deviation for triplicate analyses) as a function of the DGP concentration in the range 0 – 200 µmol/L, which dominate the mass spectra recorded during ESI titration in negative mode.

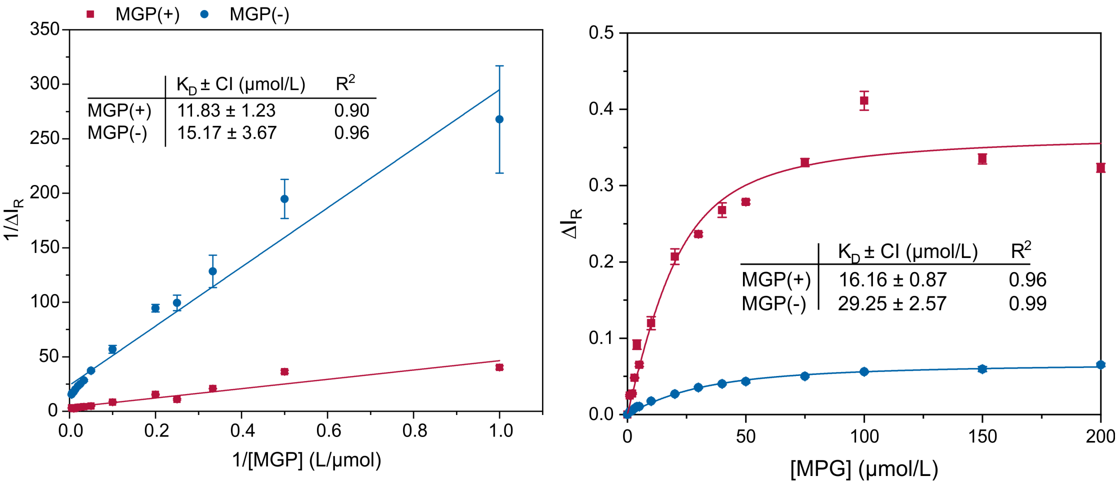

Figure S23: Left: Double reciprocal representation of the dependence of the relative intensity ΔI_R on the sugar concentration. The data were fitted linearly according to Equation 2. Right: Titration curves for the CHR:MGP complex in positive and negative ion mode. The data were fitted according to Equation 3. Error bars represent standard deviation (n = 3). The dissociation constants determined as well as their confidence interval (95 %) and the coefficient of determination of the fit function are shown in the tables.

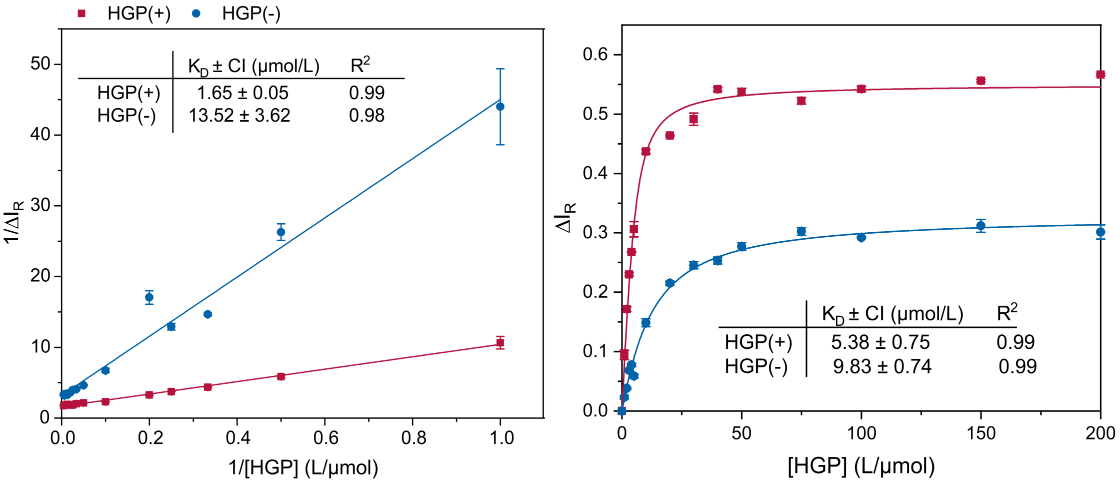

Figure S24: Left: Double reciprocal representation of the dependence of the relative intensity ΔI_R on the sugar concentration. The data were fitted linearly according to Equation 2. Right: Titration curves for the CHR:HGP complex in positive and negative ion mode. The data were fitted according to Equation 3. Error bars represent standard deviation (n = 3). The dissociation constants determined as well as their confidence interval (95 %) and the coefficient of determination of the fit function are shown in the tables.

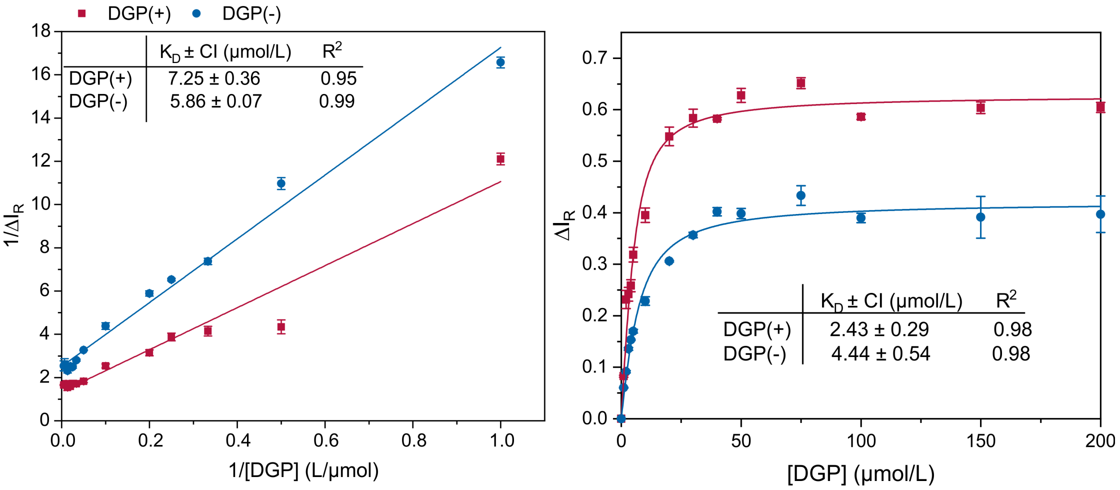

Figure S25: Left: Double reciprocal representation of the dependence of the relative intensity ΔI_R on the sugar concentration. The data were fitted linearly according to Equation 2. Right: Titration curves for the CHR:DGP complex in positive and negative ion mode. The data were fitted according to Equation 3. Error bars represent standard deviation (n = 3). The dissociation constants determined as well as their confidence interval (95 %) and the coefficient of determination of the fit function are shown in the tables.

S7. Further Results from CID-MS Experiments and CE_{50} Evaluation

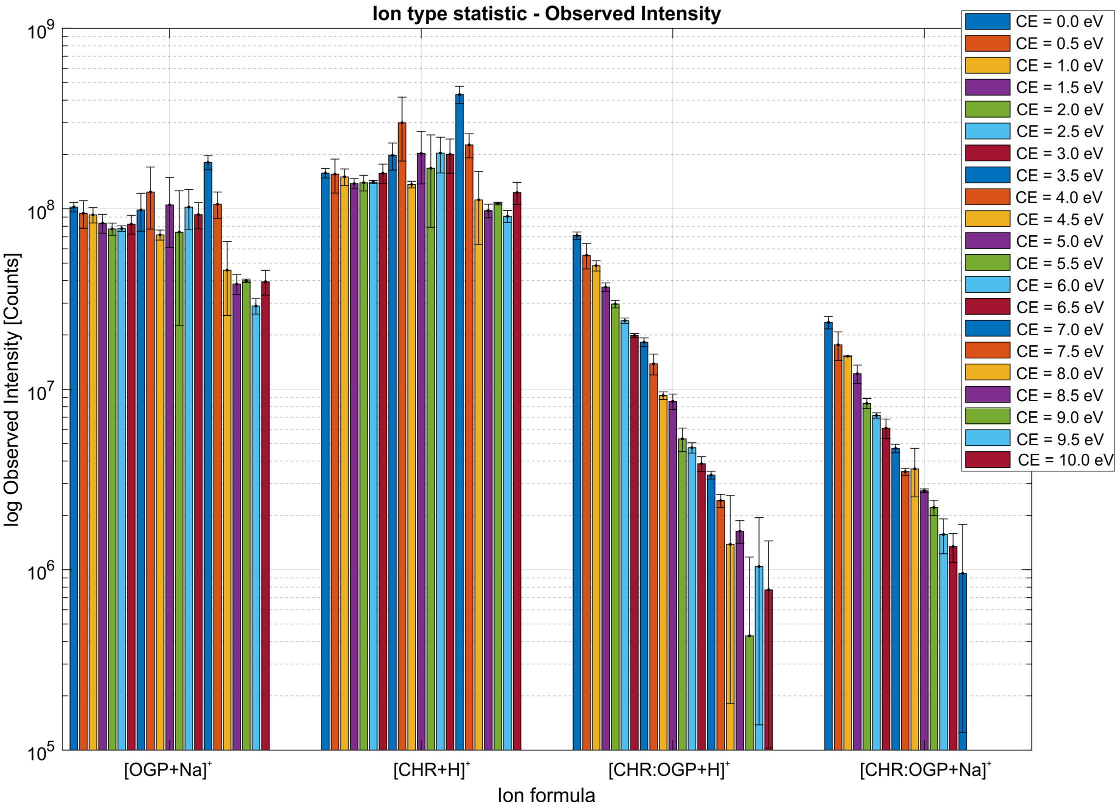

Figure S26: Observed ion abundances (positive ion mode) for the singly protonated molecular ion and the sodium adduct of the CHR:OGP complex, as well as the sodium adduct of OGP and the singly charged receptor, depending on the collision energy (CE). Error bars represent standard deviation for triplicate analyses. The measured values were used to obtain the normalized relative intensities for the breakdown curves using Equation 4. For the breakdown curves and determined CE_{50} values, see Figure 5.

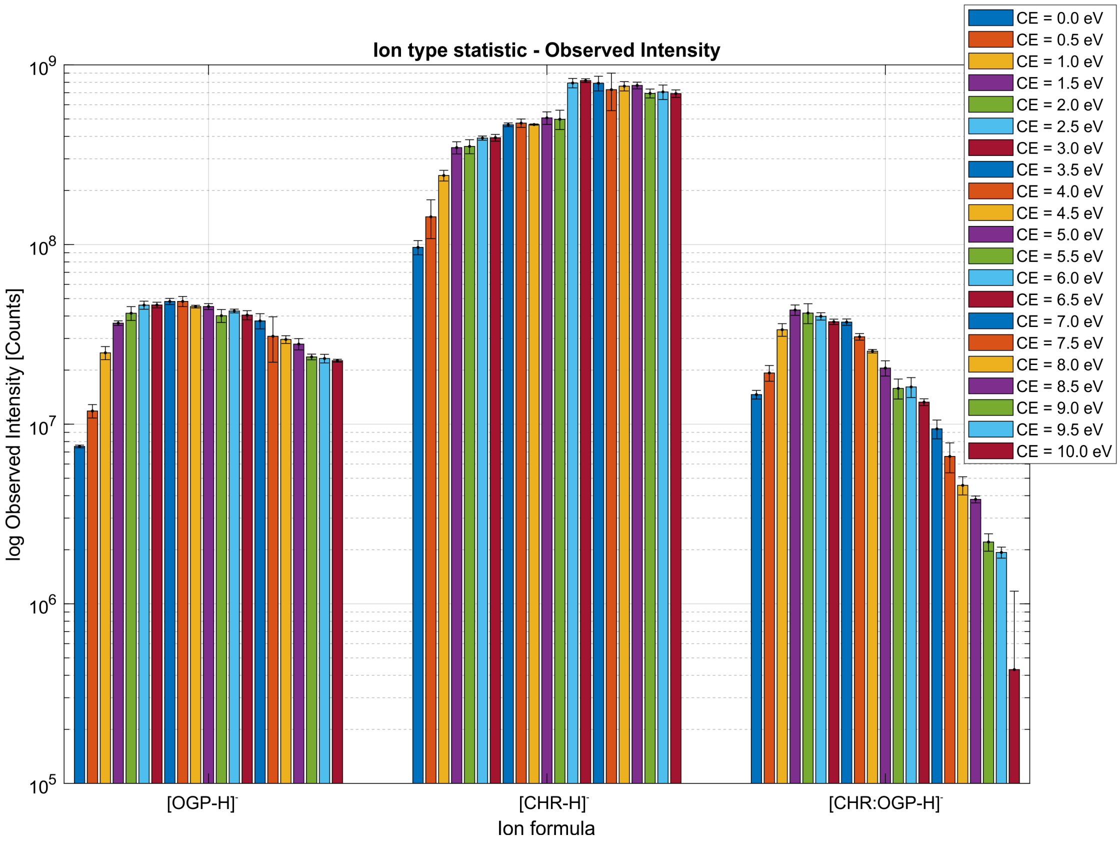

Figure S27: Observed ion abundances (negative ion mode) for the singly deprotonated molecular ion of the CHR:OGP complex as well as the fragments OGP and CHR, depending on the collision energy (CE). Error bars represent standard deviation for triplicate analyses. The measured values were used to obtain the normalized relative intensities for the breakdown curve using Equation 4. For the breakdown curve and determined CE_{50} value, see Figure 5.

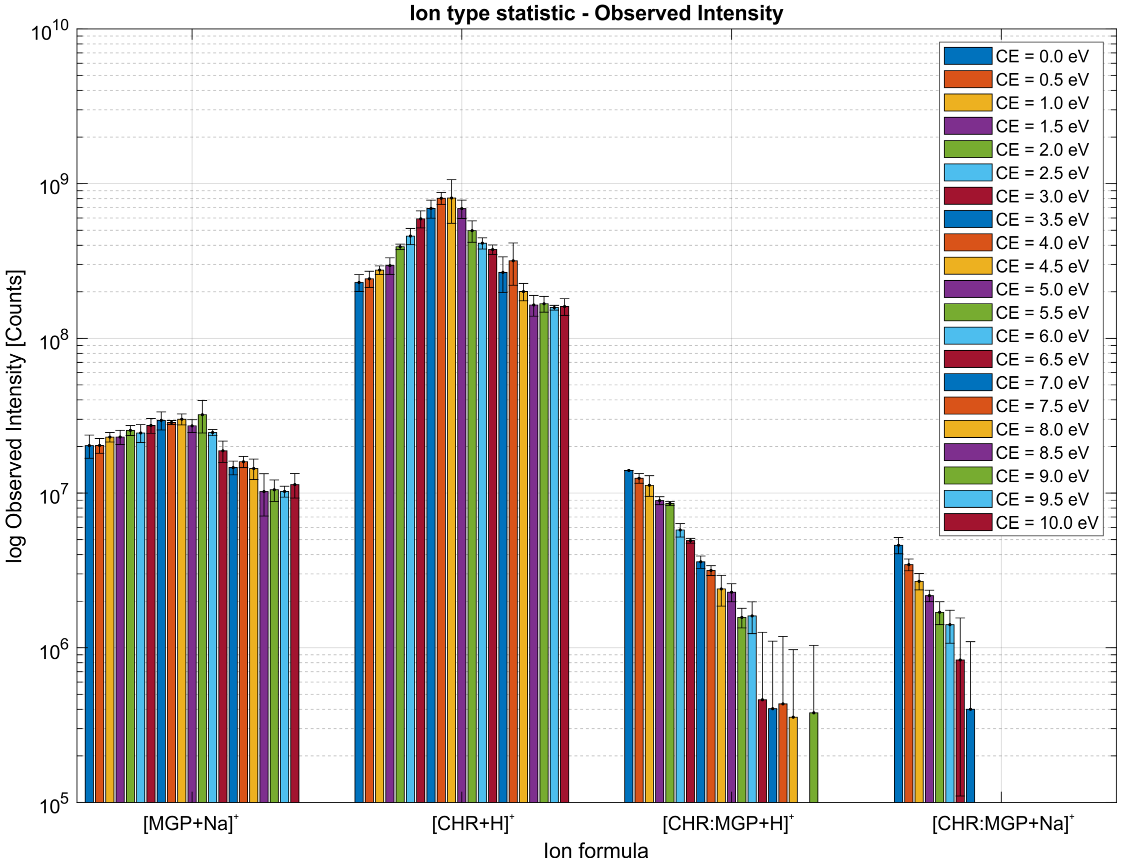

Figure S28: Observed ion abundances (positive ion mode) for the singly protonated molecular ion and the sodium adduct of the CHR:MGP complex, as well as the sodium adduct of MGP and the singly charged receptor, depending on the collision energy (CE). Error bars represent standard deviation for triplicate analyses. The measured values were used to obtain the normalized relative intensities for the breakdown curves using Equation 4. For the breakdown curves and determined CE_{50} values, see Figure S34.

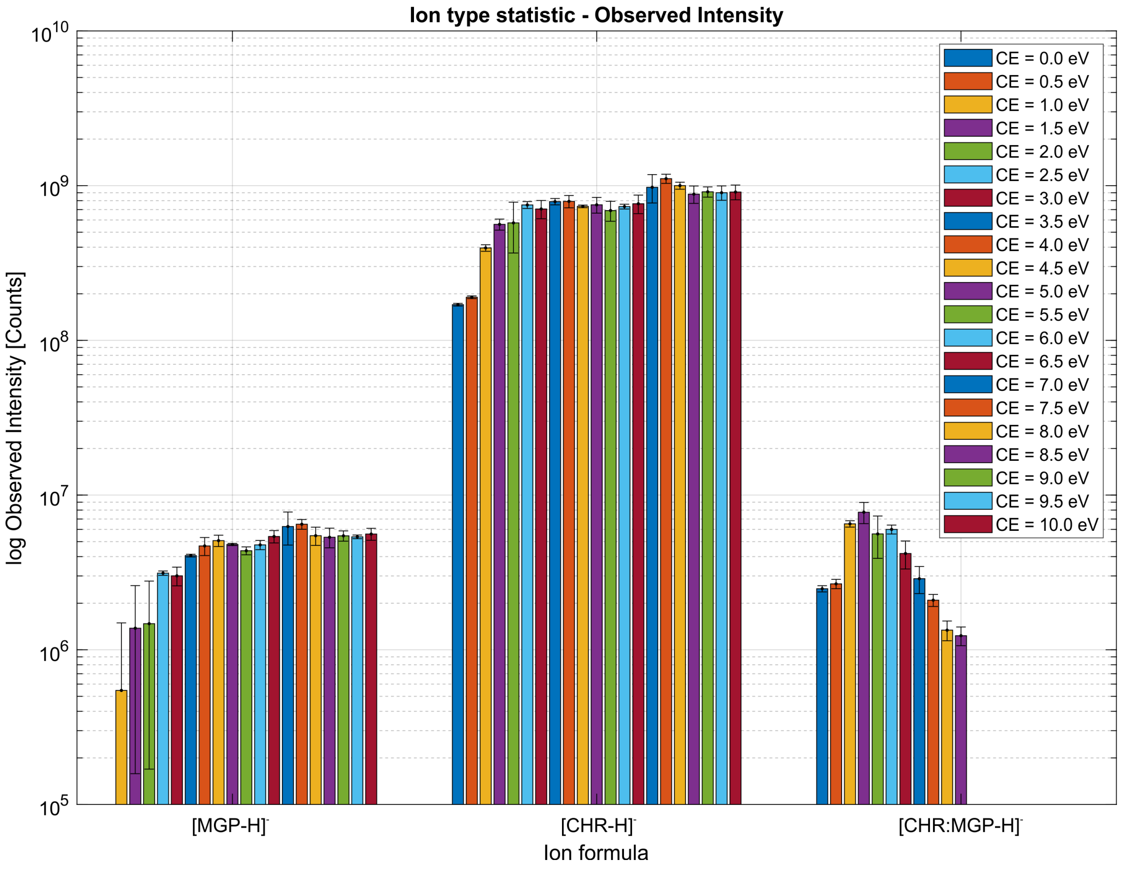

Figure S29: Observed ion abundances (negative ion mode) for the singly deprotonated molecular ion of the CHR:MGP complex as well as the fragments MGP and CHR, depending on the collision energy (CE). Error bars represent standard deviation for triplicate analyses. The measured values were used to obtain the normalized relative intensities for the breakdown curve using Equation 4. For the breakdown curve and determined CE_{50} value, see Figure S34.

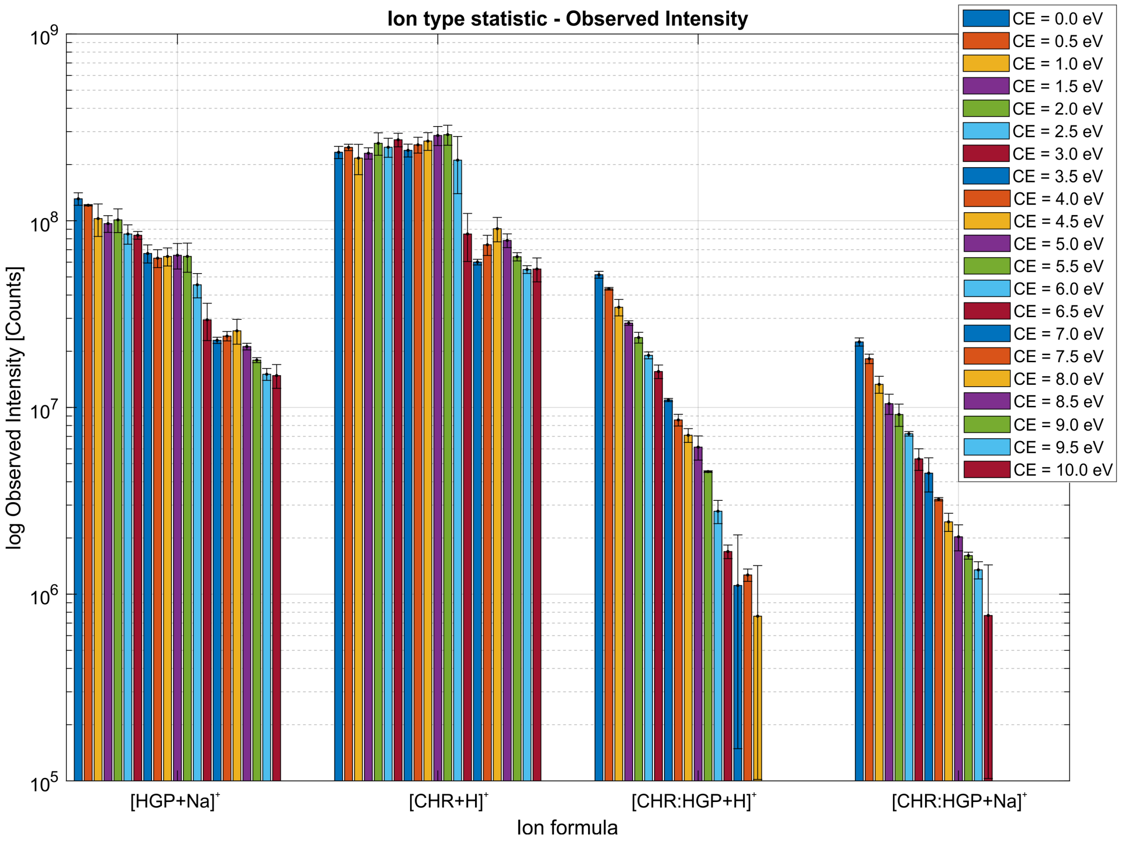

Figure S30: Observed ion abundances (positive ion mode) for the singly protonated molecular ion and the sodium adduct of the CHR:HGP complex, as well as the sodium adduct of HGP and the singly charged receptor, depending on the collision energy (CE). Error bars represent standard deviation for triplicate analyses. The measured values were used to obtain the normalized relative intensities for the breakdown curves using Equation 4. For the breakdown curves and determined CE_{50} values, see Figure S35.

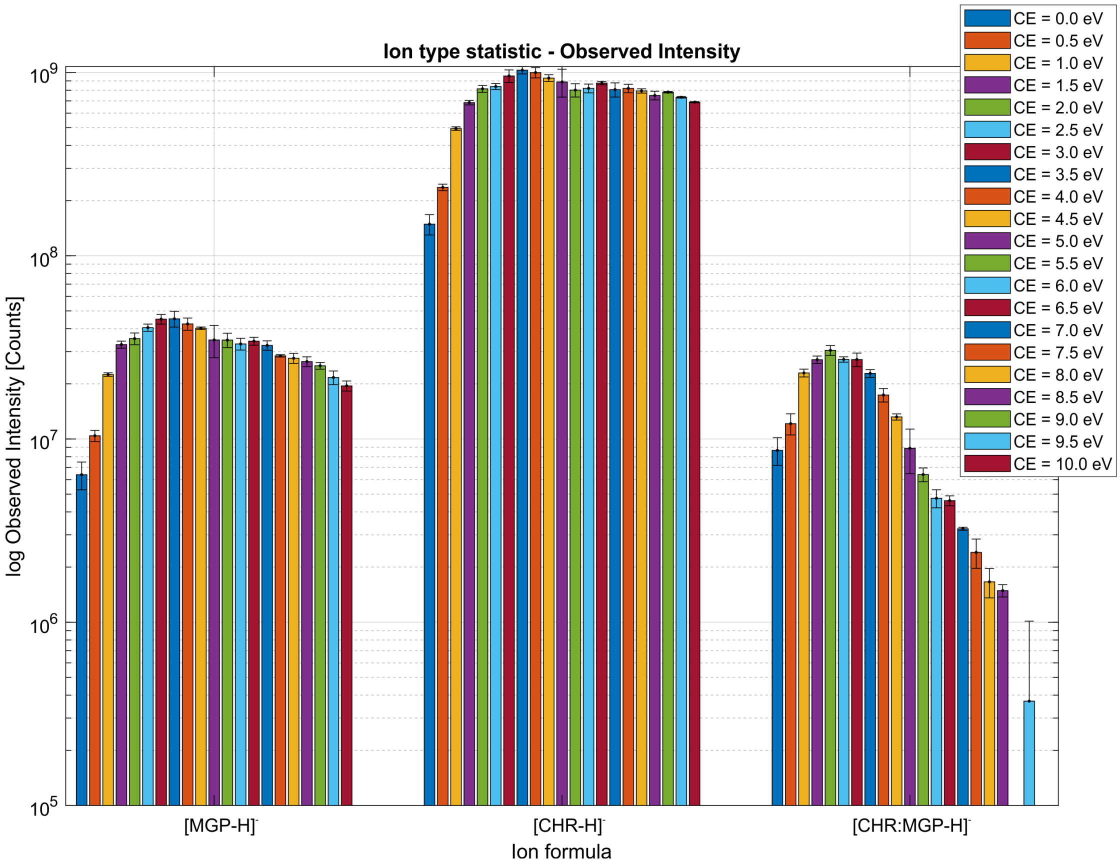

Figure S31: Observed ion abundances (negative ion mode) for the singly deprotonated molecular ion of the CHR:HGP complex as well as the fragments HGP and CHR, depending on the collision energy (CE). Error bars represent standard deviation for triplicate analyses. The measured values were used to obtain the normalized relative intensities for the breakdown curve using Equation 4. For the breakdown curve and determined CE_{50} value, see Figure S34.

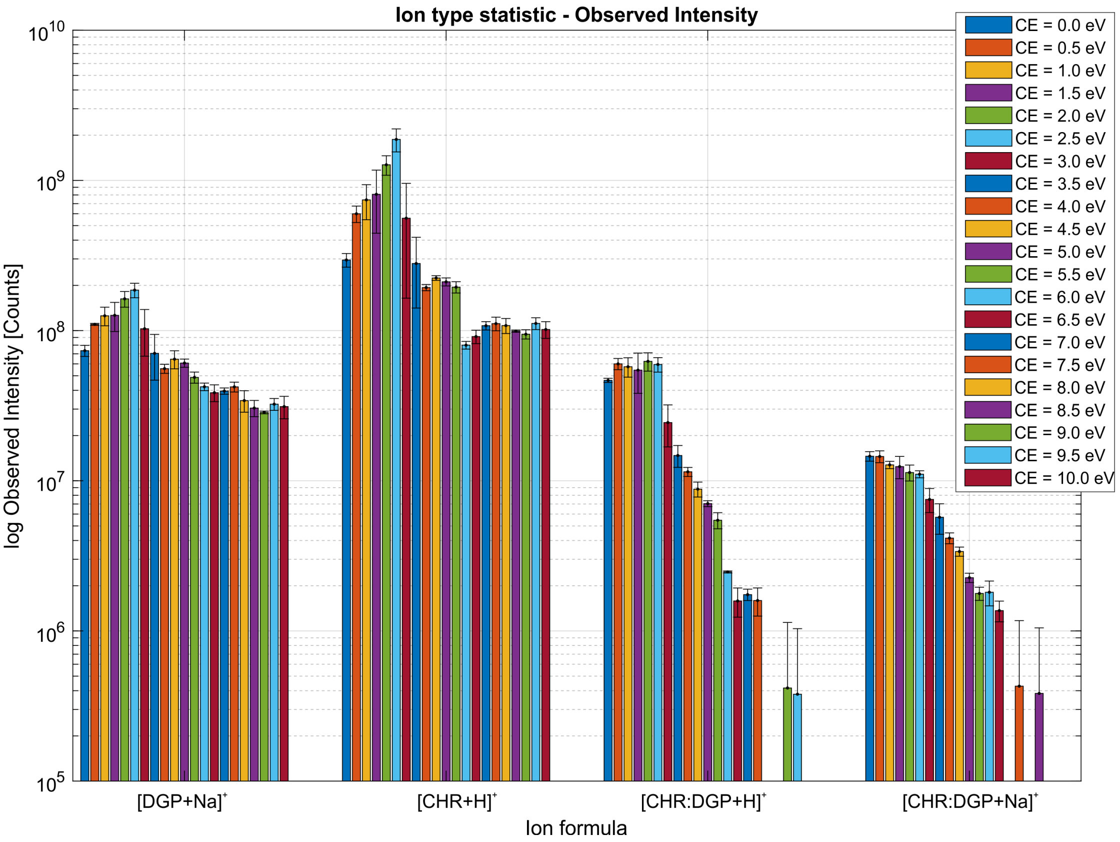

Figure S32: Observed ion abundances (positive ion mode) for the singly protonated molecular ion and the sodium adduct of the CHR:DGP complex, as well as the sodium adduct of DGP and the singly charged receptor, depending on the collision energy (CE). Error bars represent standard deviation for triplicate analyses. The measured values were used to obtain the normalized relative intensities for the breakdown curves using Equation 4. For the breakdown curves and determined CE_{50} values, see Figure S36.

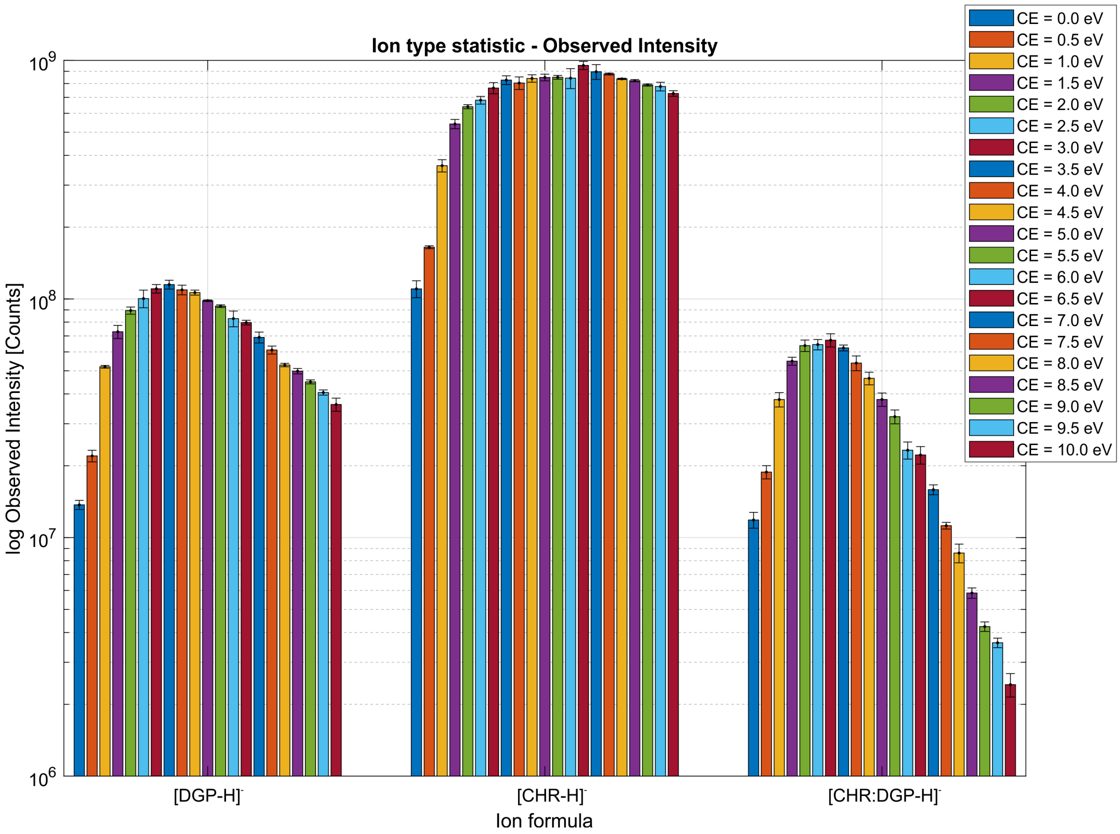

Figure S33: Observed ion abundances (negative ion mode) for the singly deprotonated molecular ion of the CHR:DGP complex as well as the fragments DGP and CHR, depending on the collision energy (CE). Error bars represent standard deviation for triplicate analyses. The measured values were used to obtain the normalized relative intensities for the breakdown curve using Equation 4. For the breakdown curve and determined CE_{50} value, see Figure S36.

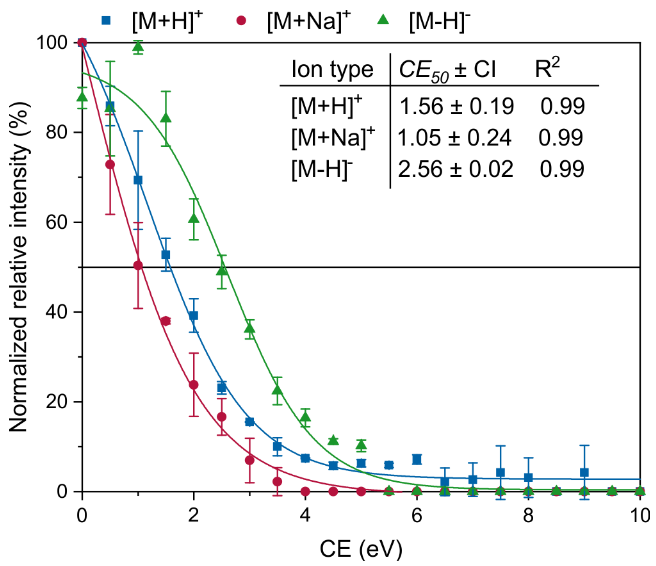

Figure S34: Normalized relative intensities of complex molecular ions $[M+H]^+$, $[M+Na]^+$ and $[M\text{-}H]^-$ for CHR:MGP as a function of the collision energy (CE) obtained in the CID-MS experiments. Error bars represent standard deviation for triplicate analyses. The CE_{50} value and the confidence interval (95 %) as well as the regression coefficient for the fit function are given for each breakdown curve.

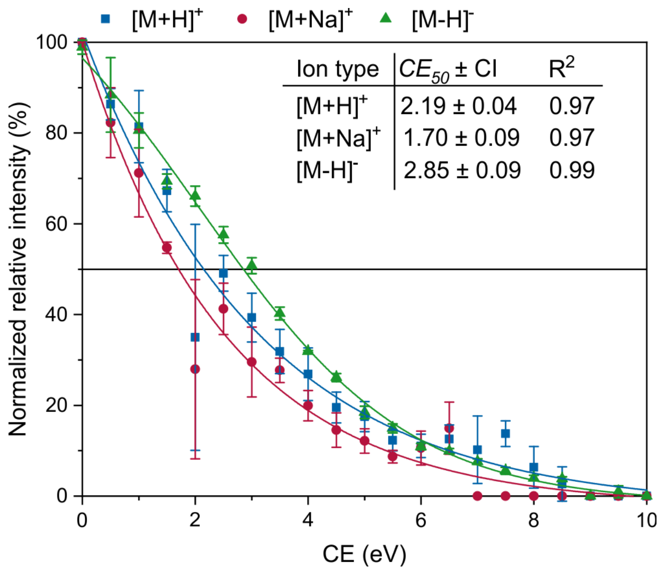

Figure S35: Normalized relative intensities of complex molecular ions $[M+H]^+$, $[M+Na]^+$ and $[M\text{-}H]^-$ for CHR:HGP as a function of the collision energy (CE) obtained in the CID-MS experiments. Error bars represent standard deviation for triplicate analyses. The CE_{50} value and the confidence interval (95 %) as well as the regression coefficient for the fit function are given for each breakdown curve.

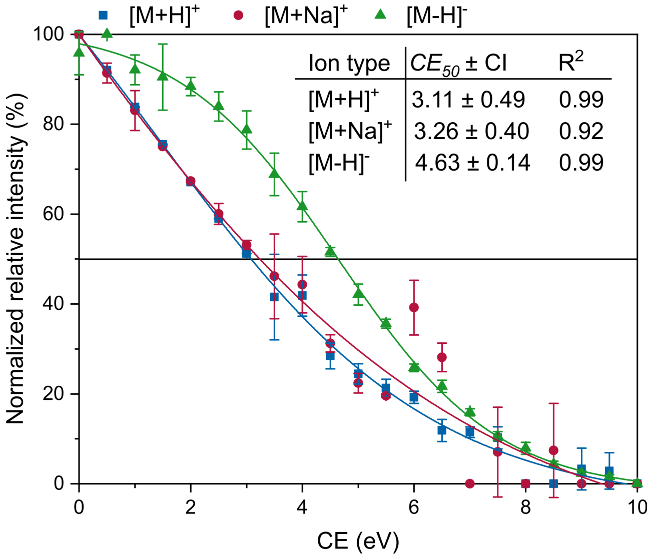

Figure S36: Normalized relative intensities of complex molecular ions $[M+H]^+$, $[M+Na]^+$ and $[M\text{-}H]^-$ for CHR:DGP as a function of the collision energy (CE) obtained in the CID-MS experiments. Error bars represent standard deviation for triplicate analyses. The CE_{50} value and the confidence interval (95 %) as well as the regression coefficient for the fit function are given for each breakdown curve.

References

(1) Dotsikas, Y.; Loukas, Y. L. Inclusional complex study between 6-p-toluidinylnaphthalene-2-sulfonate and 2-hydroxypropyl-beta-cyclodextrin. *J. Biochem. Biophys. Methods* **2002**, *52*, 121–134.

(2) Dotsikas, Y.; Loukas, Y. L. Efficient determination and evaluation of model cyclodextrin complex binding constants by electrospray mass spectrometry. *J. Am. Soc. Mass Spectrom.* **2003**, *14*, 1123–1129.

(3) Thordarson, P. Determining association constants from titration experiments in supramolecular chemistry. *Chem. Soc. Rev.* **2011**, *40*, 1305–1323.

(4) Thordarson, P. In *Supramolecular chemistry*, Gale, P. A., Steed, J. W., Eds.; Wiley: Chichester, 2012.

(5) Hirose, K. A Practical Guide for the Determination of Binding Constants. *J. Incl. Phenom. Macrocycl. Chem.* **2001**, *39*, 193–209.

(6) Hirose, K. In *Analytical Methods in Supramolecular Chemistry*; John Wiley & Sons, Ltd: 2012, pp 27–66.
